# Ferritin H Deficiency in Myeloid Compartments Dysregulates Host Energy Metabolism and Increases Susceptibility to *Mycobacterium tuberculosis* Infection

**DOI:** 10.3389/fimmu.2018.00860

**Published:** 2018-05-03

**Authors:** Vineel P. Reddy, Krishna C. Chinta, Vikram Saini, Joel N. Glasgow, Travis D. Hull, Amie Traylor, Fernanda Rey-Stolle, Miguel P. Soares, Rajhmun Madansein, Md Aejazur Rahman, Coral Barbas, Kievershen Nargan, Threnesan Naidoo, Pratistadevi K. Ramdial, James F. George, Anupam Agarwal, Adrie J. C. Steyn

**Affiliations:** ^1^Department of Microbiology, University of Alabama at Birmingham, Birmingham, AL, United States; ^2^Division of Cardiothoracic Surgery, University of Alabama at Birmingham, Birmingham, AL, United States; ^3^Nephrology Research and Training Center, University of Alabama at Birmingham and Birmingham VA Medical Center, Birmingham, AL, United States; ^4^Centro de Metabolómica y Bioanálisis (CEMBIO), Facultad de Farmacia, Universidad CEU San Pablo, Madrid, Spain; ^5^Instituto Gulbenkian de Ciência, Oeiras, Portugal; ^6^Inkosi Albert Luthuli Central Hospital, University of KwaZulu-Natal, Durban, South Africa; ^7^Africa Health Research Institute (AHRI), Durban, South Africa; ^8^Department of Anatomical Pathology, National Health Laboratory Service, University of KwaZulu-Natal, Inkosi Albert Luthuli Central Hospital, Durban, South Africa; ^9^UAB Centers for AIDS Research and Free Radical Biology, University of Alabama at Birmingham, Birmingham, AL, United States

**Keywords:** ferritin H chain, bioenergetics, immunometabolism, tuberculosis, iron, energy metabolism

## Abstract

Iron is an essential factor for the growth and virulence of *Mycobacterium tuberculosis* (*Mtb)*. However, little is known about the mechanisms by which the host controls iron availability during infection. Since ferritin heavy chain (FtH) is a major intracellular source of reserve iron in the host, we hypothesized that the lack of FtH would cause dysregulated iron homeostasis to exacerbate TB disease. Therefore, we used knockout mice lacking FtH in myeloid-derived cell populations to study *Mtb* disease progression. We found that FtH plays a critical role in protecting mice against *Mtb*, as evidenced by increased organ burden, extrapulmonary dissemination, and decreased survival in *Fth^−/−^* mice. Flow cytometry analysis showed that reduced levels of FtH contribute to an excessive inflammatory response to exacerbate disease. Extracellular flux analysis showed that FtH is essential for maintaining bioenergetic homeostasis through oxidative phosphorylation. In support of these findings, RNAseq and mass spectrometry analyses demonstrated an essential role for FtH in mitochondrial function and maintenance of central intermediary metabolism *in vivo*. Further, we show that FtH deficiency leads to iron dysregulation through the hepcidin–ferroportin axis during infection. To assess the clinical significance of our animal studies, we performed a clinicopathological analysis of iron distribution within human TB lung tissue and showed that *Mtb* severely disrupts iron homeostasis in distinct microanatomic locations of the human lung. We identified hemorrhage as a major source of metabolically inert iron deposition. Importantly, we observed increased iron levels in human TB lung tissue compared to healthy tissue. Overall, these findings advance our understanding of the link between iron-dependent energy metabolism and immunity and provide new insight into iron distribution within the spectrum of human pulmonary TB. These metabolic mechanisms could serve as the foundation for novel host-directed strategies.

## Introduction

A fundamental challenge in tuberculosis (TB) research is the identification of mechanisms whereby the disease progresses from latent to active TB. In this regard, iron has been recognized as an important factor in host immunity against bacterial infection including *Mycobacterium tuberculosis* (*Mtb*) (1–3) and plays a key role in the battle for nutritional resources between hosts and their pathogens. Several studies have shown that serum or plasma iron levels in the host modulate the pathogenicity of TB, HIV, and malaria (4–8). In addition, there is a greater risk of relapse in animal models and TB patients given iron-rich supplements (9, 10). Further, iron supplementation can greatly reduce the efficacy of anti-TB drug treatment (11). Thus, iron deficiency is a common complication in HIV and TB (12).

Humans require iron for various metabolic processes, as a cofactor for metalloenzymes, and as a key component of iron-sulfur cluster and heme-containing proteins involved in electron transport and oxidative phosphorylation (OXPHOS) in mitochondria (2, 13). Mitochondria are important signaling organelles that dictate macrophage function, which is highly integrated with cellular metabolism (14–16). M1-polarized macrophages exhibit robust glycolytic flux and reduced OXPHOS compared to M0 cells, suggesting dependence on glycolytic ATP and less reliance on mitochondrial metabolism to rapidly trigger microbicidal activity (16). This metabolic change in macrophages that accompanies the transition from M0 to M1 supports the production of metabolites, such as succinate, citrate, and NO (16, 17). Conversely, M2 polarized macrophages are associated with increased OXPHOS and changes in iron metabolism, polyamine synthesis, and fatty acid oxidation (18, 19). Here, l-arginine (Arg) metabolism plays a key role in the inflammatory function as this amino acid is required for NO production by iNOS, and by arginase-1 (Arg-1) to produce anti-inflammatory polyamines that are associated with collagen synthesis, tissue repair, cell proliferation, and memory under pathophysiological conditions (20). Arg-derived amino acids, such as Trp and Glu, are key regulators of the immune suppressive activity of myeloid-derived suppressor cells (16).

Absorption of ferrous iron occurs in the proximal duodenal epithelium *via* the divalent metal transporter 1 (21). Within the enterocyte, Fe^2+^ is bound and exported into the circulation by ferroportin (FPN), an iron exporter (22, 23). The host responds to increased systemic iron flux by secreting hepcidin (HEP), a small peptide hormone that binds to FPN and induces its internalization and degradation, thereby inhibiting iron release from enterocytes to maintain iron homeostasis (24, 25). Iron is stored intracellularly by ferritin, a major iron storage protein complex composed of 24 heavy (FtH) chains and 24 light (FtL) chains, which can bind up to 4,500 Fe^3+^ ions (26–28). In the context of iron absorption from the gut, duodenal FtH functions as a reservoir to maintain intracellular free iron at physiological levels and limit iron export to the circulation (29).

While iron acquisition and storage by *Mtb* is required to establish disease, little is known about the role of FtH in protection against bacterial infection in general, including *Mtb*, or how FtH contributes to iron homeostasis during infection (21, 30, 31). Despite elegant *in vitro* studies (30, 32–37), *in vivo* evidence for a role for host iron in TB using knockout mice has not yet been formally demonstrated, and the important link between iron and host energy metabolism has escaped the attention of many investigations. To address this gap in our knowledge, we hypothesized that dysregulation of host iron homeostasis modulates central energy metabolism and inflammation, which increases metabolic vulnerability to exacerbate TB. To test this hypothesis, we infected FtH-deficient mice with *Mtb* and assessed the role of FtH in protection against *Mtb* by examining host survival, bacillary burden, and dissemination. We used RNAseq to identify the transcriptomic signature in the lungs of infected mice and performed metabolic flux analysis to examine the role of FtH in maintaining bioenergetic homeostasis upon *Mtb* infection. Using capillary electrophoresis mass spectrometry (CE-MS) and gas chromatography mass spectrometry (GC-MS), we examined the role of FtH in central host energy metabolism. Further, we studied the link between FtH and inflammatory responses, and measured iron levels in organs of *Mtb*-infected mice. We also examined expression of iron-handling proteins in the lungs of infected *Fth^+/+^* and *Fth^−/−^* mice. Finally, we examined iron distribution in human TB lung tissues.

## Results

### *Fth^−/−^* Mice Rapidly Succumb to *Mtb* Infection

To investigate the importance of FtH during *Mtb* infection, we used mice in which FtH, a major intracellular source of reserve iron, is deleted specifically in myeloid cell populations, including monocytes/macrophages (*LyzM^cre^Fth*^Δ^*^/^*^Δ^, hereafter referred to as *Fth^−/−^*). Control mice expressing FtH (*Fth^Flox/Flox^*, hereafter referred to as *Fth*^+/+^) express normal levels of FtH and are homozygous for a floxed allele and express FtH in all myeloid cells. We confirmed deletion of the *Fth* allele in *Fth^−/−^* mice by PCR (Figure S1A in Supplementary Material) and immunoblotting (Figure S1B in Supplementary Material) of bone marrow-derived macrophage (BMDM) lysates. Expression of FtL was increased in *Fth^−/−^* macrophages compared to those from *Fth^+/+^* mice (Figure S1B in Supplementary Material).

**Figure 1:**
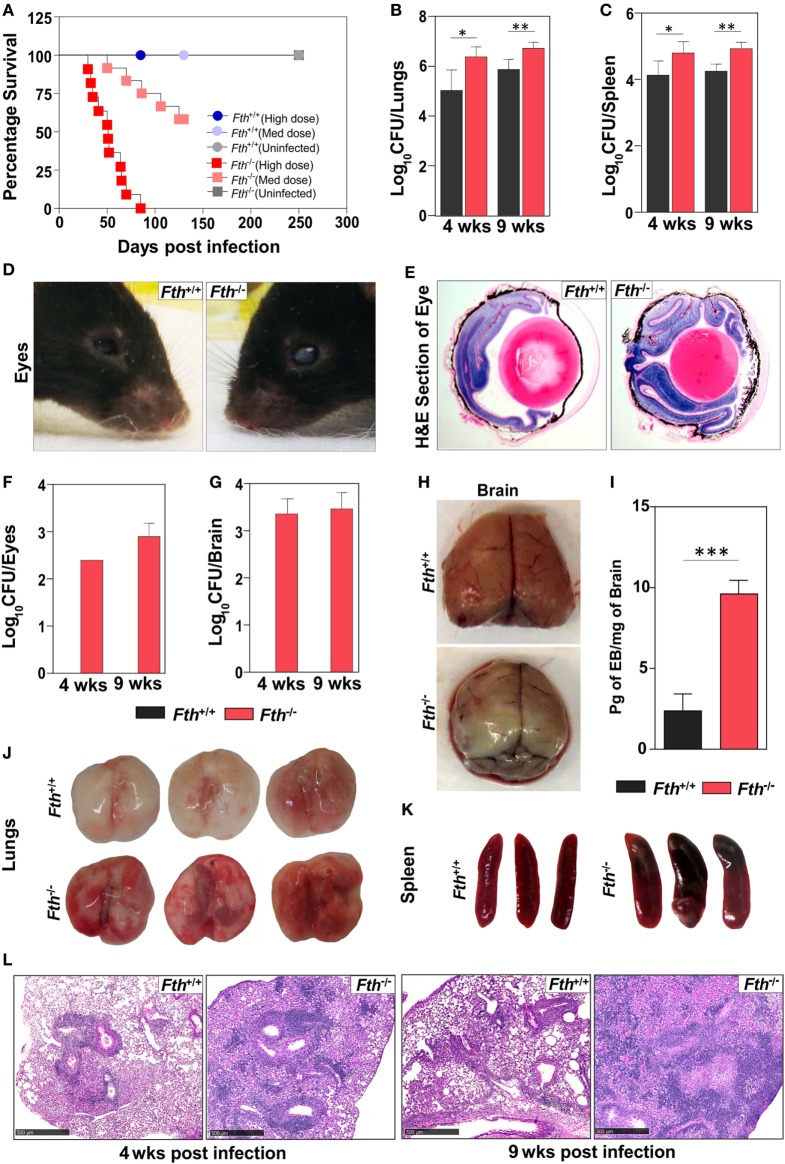
Survival, bacillary burden, and pathology of mice following *Mtb* infection. **(A)** Kaplan–Meier survival curve showing the death of *Fth^−/−^* mice following high dose and medium dose of *Mtb* infection (*n* = 11). **(B,C)** Bacillary burden in the lungs and spleens of mice at 4 and 9 weeks postinfection (*n* = 7). **(D)** Representative photograph of mouse eyes at 9 weeks postinfection (*n* = 5). **(E)** Representative H&E staining of eyes showing more vasculitis in *Fth^−/−^* mice compared to *Fth^+/+^* mice (*n* = 5). **(F,G)** Bacillary burden in the eyes and brains of mice at 4 and 9 weeks postinfection (*n* = 7). **(H)** Representative photograph of brains from *Mtb*-infected mice 30 min after i.v. injection of Evans blue dye (*n* = 5). **(I)** Quantitation of Evans blue dye extracted from the brains of infected mice (*n* = 5). **(J,K)** Gross pathology of lungs and spleen of mice at 4 weeks postinfection (*n* = 5). **(L)** Representative micrographs of H&E staining of lungs from mice at 4 and 9 weeks postinfection (*n* = 7). Peribronchial and perivascular aggregation of histiocytes and the position of fibrous tissues in tuberculosis lesions in wild-type and knockout mice at 4 and 9 weeks postinfection. Scale bar; 500 µm. Statistical testing was performed using the unpaired Student’s *t*-test. Data are represented as mean ± SEM (*p < 0.05, **p < 0.01, ***p < 0.001).

To determine the role of FtH during *Mtb* infection, we infected *Fth^+/+^* and *Fth^−/−^* mice with a high dose of *Mtb* (~2,500 cfu/animal) and noted that *Fth^−/−^* mice are highly susceptible to TB disease and died by 85 days postinfection (mean survival time: 51 days). With a medium dose of *Mtb* (~300 cfu/animal), only five mice died by day 130 postinfection, showing that *Fth^−/−^* mice have much higher susceptibility to *Mtb* infection (Figure 1A). No *Fth^+/+^* mice died with either dose of *Mtb* infection. The medium dose of ~300 cfu/animal was used for all subsequent *Mtb* infections. At 4 and 9 weeks postinfection, the bacillary burden in the lungs and spleens of *Fth^−/−^* mice was over fivefold higher than in *Fth^+/+^* mice (Figures 1B,C). Interestingly, we observed whitening and shrinking leading to blindness of the eyes in infected *Fth^−/−^* mice by 6 weeks postinfection (Figure 1D). H&E staining of eye sections showed more retinal cuffing (vasculitis) in infected *Fth^−/−^* mice compared to *Fth^+/+^* mice (Figure 1E). *Mtb* bacilli were recovered from eyes of *Fth^−/−^* mice whereas none were detected in *Fth^+/+^* mice (Figure 1F). To further investigate dissemination, we determined the bacillary burden in the brain. We recovered *Mtb* bacilli from the brains of *Fth^−/−^* mice, but not *Fth^+/+^* mice at 4 and 9 weeks postinfection (Figure 1G). To evaluate the integrity of the blood–brain barrier in *Mtb*-infected mice, we administered Evans blue dye (EB) intravenously 4 weeks postinfection. We observed blue staining in the brains of the *Fth^−/−^* mice, whereas no staining was seen in *Fth^+/+^* mice (Figure 1H). Further, quantitation of EB extracted from brain tissue revealed significantly higher amounts in *Fth^−/−^* mice compared to *Fth^+/+^* (Figure 1I). These findings substantiate the disruption of the blood–brain barrier in *Mtb*-infected *Fth^−/−^* mice.

Examination of gross organ pathology revealed an increased number of large lesions in the lungs and splenomegaly in *Fth^−/−^* mice at 4 weeks postinfection (Figures 1J,K). Histopathological analysis revealed numerous tuberculous lesions composed of perivascular and peribronchial histiocytic aggregates that were more consolidated at 9 weeks than at 4 weeks and worse in *Fth^−/−^* than *Fth^+/+^* mice. Evidence of hemorrhage was also more pronounced in *Fth^−/−^* mice with loss of lung architecture at 9 weeks postinfection (Figure 1L). In contrast, *Fth^+/+^* mice exhibited markedly better lung alveolar space at 4 and 9 weeks postinfection (Figure 1L). Overall, the organ burden, survival, and pathology data provide *in vivo* evidence that FtH is crucial for host protection against *Mtb* disease progression.

### *Mtb* Infection Dysregulates Iron Homeostasis in *Fth^−/−^* Mice

**Figure 2:**
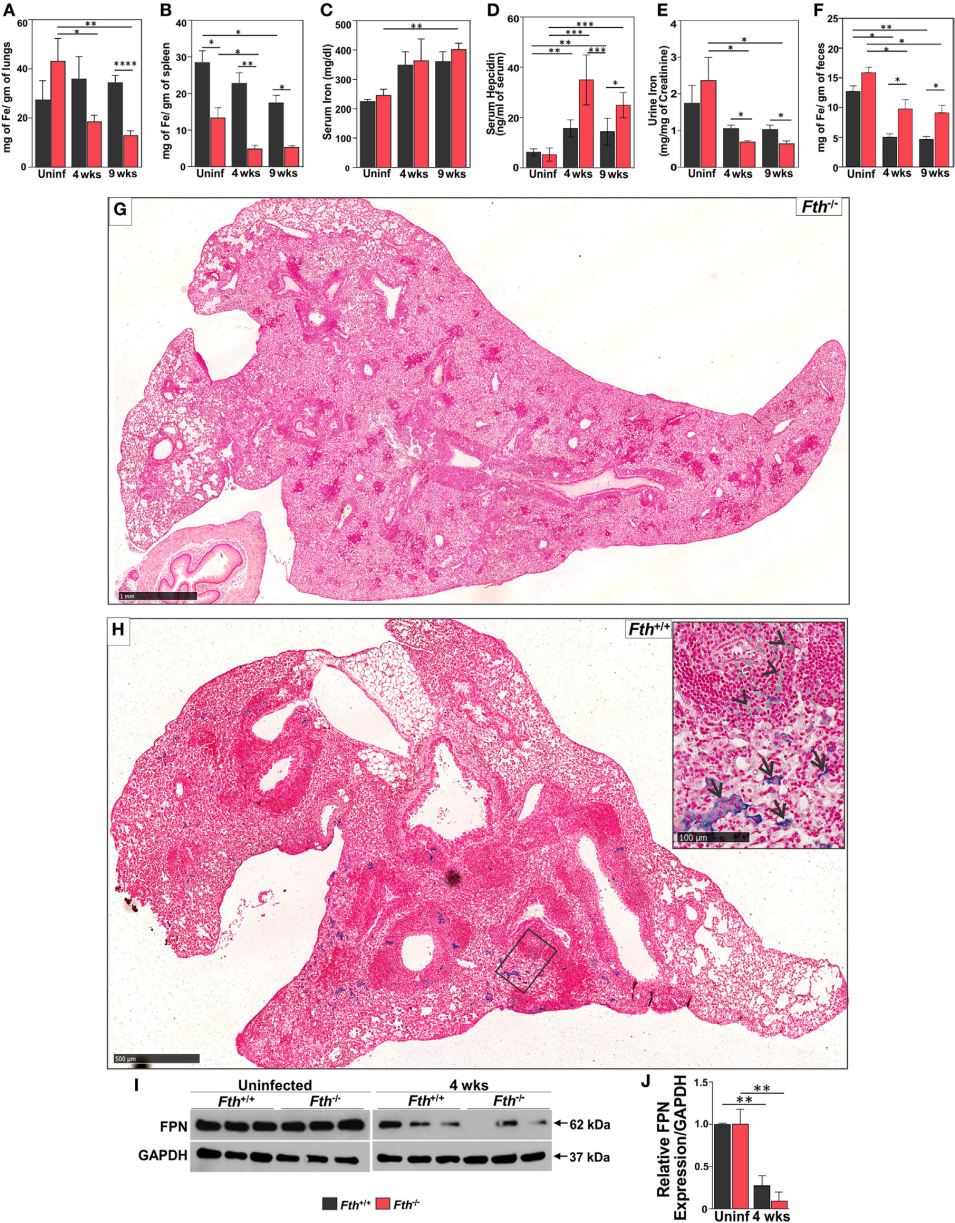
Quantification of iron in mouse tissues following *Mtb* infection. **(A–C)** Iron levels in lungs, spleens, and sera of *Fth^+/+^* and *Fth^−/−^* mice by inductively coupled plasma mass spectrometry (*n* = 7). **(D)** Hepcidin levels in the sera of mice at 4 and 9 weeks postinfection (*n* = 7). **(E)** Iron levels in urine of mice at 4 and 9 weeks postinfection (*n* = 7). Iron levels in urine were normalized to creatinine. **(F)** Iron levels in feces of mice at 4 and 9 weeks postinfection (*n* = 7). **(G)** Iron Von Giesen stain demonstrating minimal hemosiderin deposition in an *Fth^−/−^* mouse lung, representative of (*n* = 6). **(H)** Conspicuous hemosiderin deposition in an *Fth^+/+^* mouse lung, representative of (*n* = 6). Inset: high-magnification iron Von Giesen stain demonstrating blue-green siderophages (arrows) and foci of hemorrhage (arrowheads) in *Fth^+/+^* mouse. **(I)** Western blot showing ferroportin (FPN) in the duodenum of three mice at 4 weeks postinfection. **(J)** Densitometry analysis of relative FPN expression in duodenum. Expression of iron related proteins in lungs following *Mtb* infection. Statistical testing was performed using the unpaired Student’s *t*-test. Data are represented as mean ± SEM (*p < 0.05, **p < 0.01, ***p < 0.001, ****p < 0.0001).

Increased free circulating iron in the host supports rapid growth of *Mtb* and worsens the outcome of TB disease in humans and mice (9). Reduction of iron availability is a host defense mechanism against *Mtb* infection that can result in anemia in humans (12). Since FtH reduces the availability of free iron (38), we anticipated that a lack of FtH in myeloid cells would result in increased levels of free iron leading to increased mortality of *Mtb*-infected *Fth^−/−^* mice (Figure 1A). However, iron levels were lower in the lungs and spleens of *Fth^−/−^* mice compared to *Fth*^+/+^ mice at 4 and 9 weeks postinfection (Figures 2A,B). This was confirmed by Prussian blue staining, which shows striking evidence of iron deposition in the lungs of *Fth^+/+^*, but not *Fth^−/−^*, mice at 9 weeks postinfection (Figures 2G,H).

Total iron in the lungs of uninfected *Fth^+/+^* and *Fth^−/−^* mice was not significantly different. As shown in Figure 2C, total iron levels in the serum of *Fth^+/+^* and *Fth^−/−^* mice were increased at 4 and 9 weeks postinfection compared to uninfected. Despite similar iron levels in the serum, *Mtb*-infected *Fth^−/−^* mice had significantly increased HEP levels compared to *Fth^+/+^* mice (Figure 2D), suggesting that iron homeostasis in *Fth^−/−^* mice is more dysregulated than in *Fth^+/+^* mice. We observed reduced levels of urinary iron in *Fth^−/−^* mice compared to *Fth^+/+^* mice at both 4 and 9 weeks following *Mtb* infection (Figure 2E). To evaluate absorption of iron from the gut, we measured total iron in feces and observed significantly higher iron levels in the feces of *Fth^−/−^* mice, indicating reduced iron uptake (Figure 2F). Finally, we examined FPN expression in the duodenum of *Fth^−/−^* and *Fth^+/+^* mice to assess whether iron export from the gut is impaired in *Fth^−/−^* mice upon *Mtb* infection. No difference was seen in duodenal FPN expression between uninfected *Fth^−/−^* and *Fth^+/+^* mice (Figures 2I,J). However, *Mtb* infection led to a marked reduction in FPN expression in both *Fth^−/−^* and *Fth^+/+^* mice, although *Fth^+/+^* infected mice consistently expressed slightly more FPN (Figure 2I). Overall, these data demonstrate that in the absence of myeloid FtH, host iron homeostasis is disrupted as evidenced by decreased iron in the lung and spleen, increased HEP levels, reduced duodenal FPN, and reduced iron absorption from the gut.

### Iron-Associated Proteins in Lungs Are Upregulated in *Fth^−/−^* Mice Upon *Mtb* Infection

**Figure 3:**
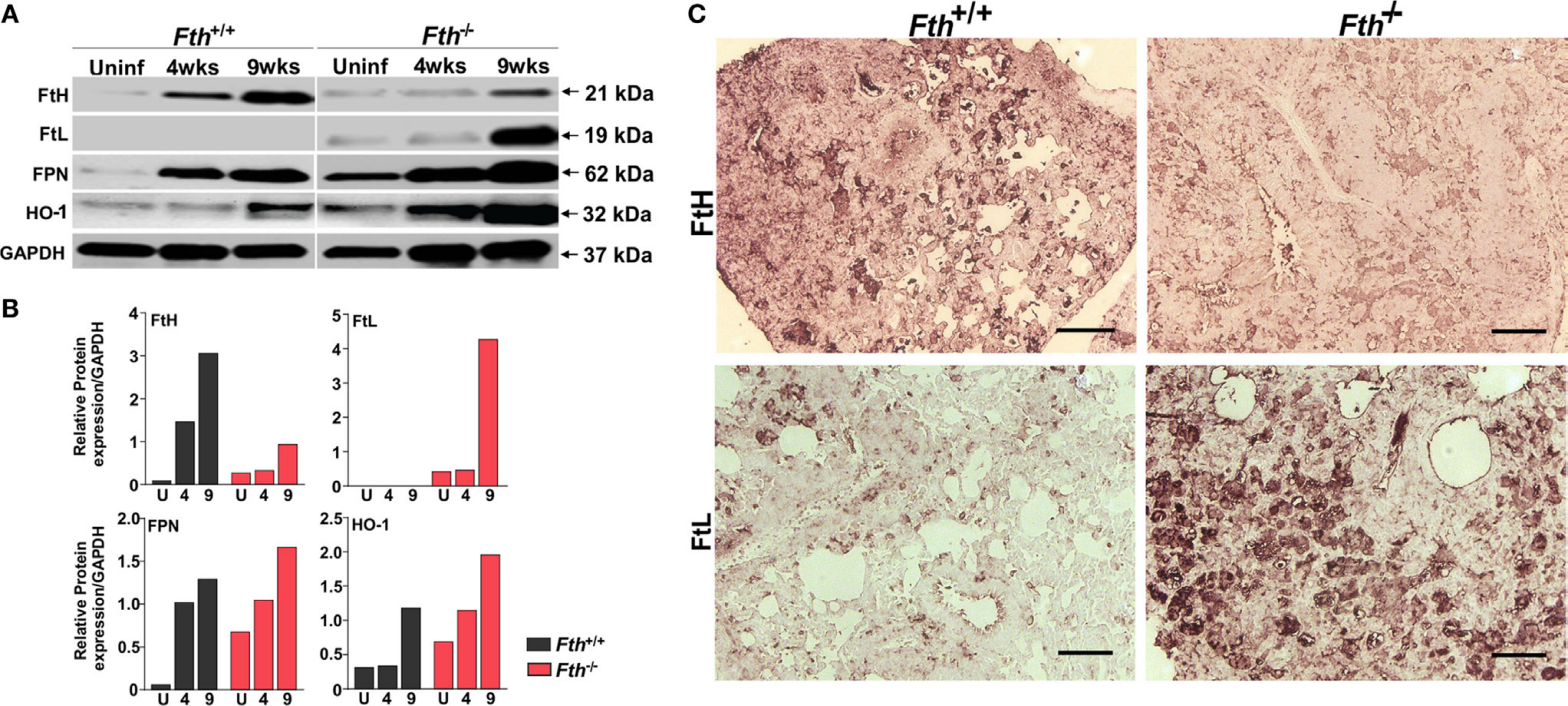
Expression of iron related proteins in mouse lungs following *Mtb* infection. **(A)** Western blot showing expression of FtH, FtL, ferroportin (FPN), and heme oxygenase-1 (HO-1) in the lungs of *Fth^+/+^* and *Fth^−/−^* mice in uninfected animals and at 4 and 9 weeks postinfection (pooled samples, *n* = 7). **(B)** Densitometric quantitation of western blot bands in panel **(A)** shown as relative protein expression normalized to GAPDH band intensity. **(C)** Representative image of immunohistochemical analysis of FtH and FtL expression in the lungs of mice at 9 weeks postinfection (*n* = 7). Scale bar; 100 µm.

Next, to better understand the role of iron-associated proteins in lungs during infection and why iron levels are reduced in the lungs of *Fth^−/−^* mice, we examined the expression of ferritin heavy chain (FtH), ferritin light chain (FtL), the iron exporter FPN, and heme oxygenase-1 (HO-1) by immunoblot. HO-1 is a cytoprotective enzyme which is induced during *Mtb* infection and uses heme as substrate to release iron, which is strongly bound by FtH. FtH expression in uninfected *Fth^+/+^* mice was barely detectable; however, infection markedly increased FtH production. As expected, total FtH in the lungs of *Fth^−/−^* mice was lower after infection due to the lack of FtH expression in macrophages (Figures 3A–C). Notably, we observed increased FtL levels in the lungs of *Mtb*-infected *Fth^−/−^* mice at 9 weeks postinfection (Figures 3A,B) which is likely a compensatory response to limiting FtH levels. Compared to *Fth^+/+^* mice, we observed higher levels of FPN in the lungs of uninfected *Fth^−/−^* mice, which increased following *Mtb* infection (Figures 3A,B). This is consistent with the reduced levels of iron in these lungs (Figure 2A). HO-1 levels in *Fth^−/−^* lungs were higher than in *Fth^+/+^* after infection (Figures 3A,B), which suggests increased heme catabolism and is consistent with increased FPN (Figures 3A,B). These data suggest that in the absence of myeloid FtH, the heme-degrading enzyme HO-1 is upregulated with the concomitant release of iron. The host responds to the increased iron levels by pronounced overexpression of iron-handling (FtL) and iron export (FPN) proteins, leading to reduced levels of iron in the lungs of *Fth^−/−^* mice.

### *Fth^−/−^* Mice Exhibit Stronger Th-1 Responses Following *Mtb* Infection

**Figure 4:**
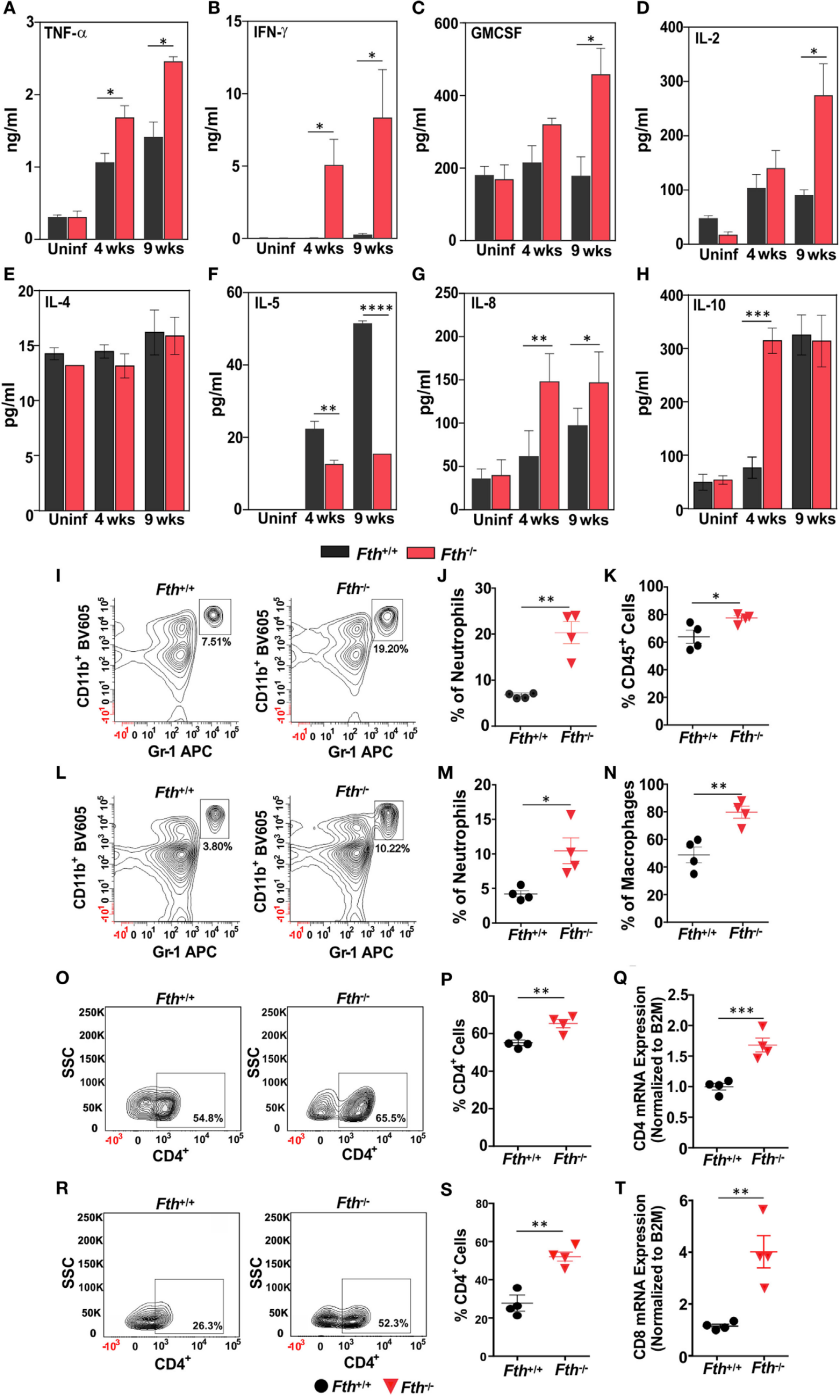
*Fth^−/−^* mice elicited strong Th-1 response upon *Mtb* infection. **(A–H)** Levels of TNF-α, IFN-γ, GM-CSF, IL-2, IL-4, IL-5, IL-8, and IL-10 in bronchoalveolar lavage fluid obtained from uninfected *Fth^+/+^* and *Fth^−/−^* mice and at 4 and 9 weeks postinfection (*n* = 7). **(I)** Representative contour plots showing the percent of CD11b^+^/Gr-1(1A8)^bright^ neutrophils (box) isolated from lungs 4 weeks postinfection. **(J)** Percentage of neutrophils in the lungs of mice at 4 weeks postinfection (*n* = 4). **(K)** Percentage of CD45^+^ cells in the lungs of mice at 4 weeks postinfection (*n* = 4). **(L)** Representative contour plots showing the percent of CD11b^+^/Gr-1(1A8)^bright^ neutrophils (box) isolated from spleens 4 weeks postinfection. **(M)** Percentage of neutrophils in spleens of mice at 4 weeks postinfection (*n* = 4). **(N)** Percentage of CD11b^+^/ly6C^+^ macrophages in the spleen of mice at 4 weeks postinfection (*n* = 4). **(O)** Representative contour plots showing the percent of CD4^+^ cells (box) from the lungs of mice at 4 weeks postinfection. **(P)** Percentage of CD4^+^ cells in the lungs of mice at 4 weeks postinfection (*n* = 4). **(Q)** Relative expression of CD4 mRNA levels in the lungs of *Fth^+/+^* and *Fth^−/−^* mice at 4 weeks postinfection (*n* = 4). Expression levels were normalized to β-2 microglobulin. **(R)** Representative contour plots showing percentage of CD4^+^ cells (box) from the spleens of mice at 4 weeks postinfection. **(S)** Percentage of CD4^+^ cells in the spleens of mice at 4 weeks postinfection (*n* = 4). **(T)** Relative expression of CD8 mRNA levels in the lungs of *Fth^+/+^* and *Fth^−/−^* mice at 4 weeks postinfection (*n* = 4). Expression levels were normalized to β-2 microglobulin. Statistical testing was performed using the unpaired Student’s *t*-test. Data are represented as mean ± SEM (*p < 0.05, **p < 0.01, ***p < 0.001, ****p < 0.0001).

To identify the immunological mechanisms responsible for increased susceptibility to *Mtb*, we hypothesized that the loss of myeloid FtH leads to enhance innate immune responses to *Mtb* infection. We measured cytokine levels in bronchoalveolar lavage (BAL) fluid from *Fth^+/+^* and *Fth^−/−^* mice. Levels of Th-1 cytokines IFN-γ, TNF-α, IL-2, and GM-CSF were significantly higher in *Fth^−/−^* mice compared to *Fth^+/+^* mice following *Mtb* infection (Figures 4A–D). In contrast, the Th-2 cytokine IL-5 was significantly downregulated in *Fth^−/−^* mice compared to *Fth^+/+^* (Figure 4F), whereas IL-8 levels were elevated in *Fth^−/−^* mice compared to *Fth^+/+^* following *Mtb* infection (Figure 4G). This suggests that IL-5 may regulate IL-8 levels in *Fth^+/+^* and *Fth^−/−^* mice at both 4 and 9 weeks postinfection (Figure 4F). IL-10 was highly upregulated at 4 weeks in *Fth^−/−^* mice, but levels were similar to *Fth^+/+^* mice at 9 weeks postinfection (Figure 4H). IL-4 levels were not significantly different between uninfected *Fth^+/+^* and *Fth^−/−^* mice, or at 4 and 9 weeks postinfection (Figure 4E). No significant differences in cytokine levels were observed between uninfected *Fth^+/+^* and *Fth^−/−^* mice, and IL-5 was not detected in uninfected mice. The cytokines IL-1, IL-6, IL-12 are known to have important roles in pathogenesis and host defense during *Mtb* infection (39). We, therefore, measured IL-1α, IL-3, IL-6, IL-12, IL-17, eotoxin, G-CSF, KC, RANTES, MIP-1α, and MIP-1β in the BAL fluid of *Mtb*-infected mice. We observed that all these cytokines were significantly upregulated in *Mtb*-infected *Fth^−/−^* mice compared to *Fth^+/+^* mice (Figures S2A–K in Supplementary Material).

Leukocytes expressing CD45^+^ accumulate at the site of lung injury (40). We observed a significantly higher percentage of CD45^+^ cells in the lungs of *Fth^−/−^* mice compared to *Fth^+/+^* mice (Figure 4K). Neutrophils and macrophages are major effector cells of innate immunity which phagocytose and kill *Mtb* (41). We next quantified neutrophils and macrophages isolated from the lungs of infected *Fth^−/−^* and *Fth^+/+^* mice. At 4 weeks postinfection, we observed significantly more neutrophils (CD11b^+^/GR-1^+^) in the lungs of *Mtb-*infected *Fth^−/−^* mice (19.2%) Vs *Fth^+/+^* (7.5%) (Figures 4I,J). Similarly, we observed significantly more neutrophils in the spleens of *Fth^−/−^* mice (10.2%) compared to *Fth^+/+^* mice (3.8%) (Figures 4L,M). Further, significantly more macrophages (CD11b^+^/Ly6C^+^) were observed in spleens of *Fth^−/−^* mice compared to *Fth^+/+^* (Figure 4N). At 9 weeks postinfection, we observed more neutrophils in the lungs of *Mtb*-infected *Fth^−/−^* mice (14.9%) compared to *Fth^+/+^* mice (4.8%) (Figures S3A–C in Supplementary Material). An increased number of neutrophils and macrophages were observed in spleens of *Fth^−/−^* mice (11.2%) compared to *Fth^+/+^* mice (7.4%) (Figures S3D–F in Supplementary Material). We observed no difference in the percentages of CD45^+^ cells and neutrophils in the lungs of uninfected *Fth^−/−^* and *Fth^+/+^* mice (Figures S3G–I in Supplementary Material). We also observed similar percentages of macrophages (CD11b^+^/Ly6C^+^) in the lungs of *Fth^−/−^* and *Fth^+/+^* mice at both 4 and 9 weeks postinfection (Figures S3J,K in Supplementary Material).

At 4 weeks postinfection, the percentage of CD4^+^ cells were increased in the lungs of *Fth^−/−^* mice (65.5%) compared to *Fth^+/+^* mice (54.8%) (Figures 4O,P). To confirm this observation, we quantified CD4 mRNA in the lungs of infected mice. As shown in Figure 4Q, the lungs of *Fth^−/−^* mice have significantly higher CD4 mRNA expression compared to *Fth^+/+^* mice at 4 weeks postinfection. Similarly, we observed significantly more CD4^+^ cells in the spleens of infected *Fth^−/−^* mice (52.3%) compared to *Fth^+/+^* mice (26.3%) (Figures 4R,S). Further, qRT-PCR revealed significantly increased CD8 mRNA levels in the lungs of *Fth*-deficient mice compared to *Fth^+/+^* (Figure 4T).

In sum, our data show that reduced myeloid FtH leads to enhanced immune cell infiltration into the lungs, thereby contributing to a significantly increased pro-inflammatory response to *Mtb* and a diminished ability to control infection. The initial IL-10-mediated anti-inflammatory response was rapidly overwhelmed at the later stage of infection. Overall, the data show that following infection, dysregulation of FtH-mediated iron homeostasis in myeloid cells contributes toward excessive inflammatory response *via* Th1 cytokines.

### FtH Is Necessary for Sustaining Immunometabolism

**Figure 5:**
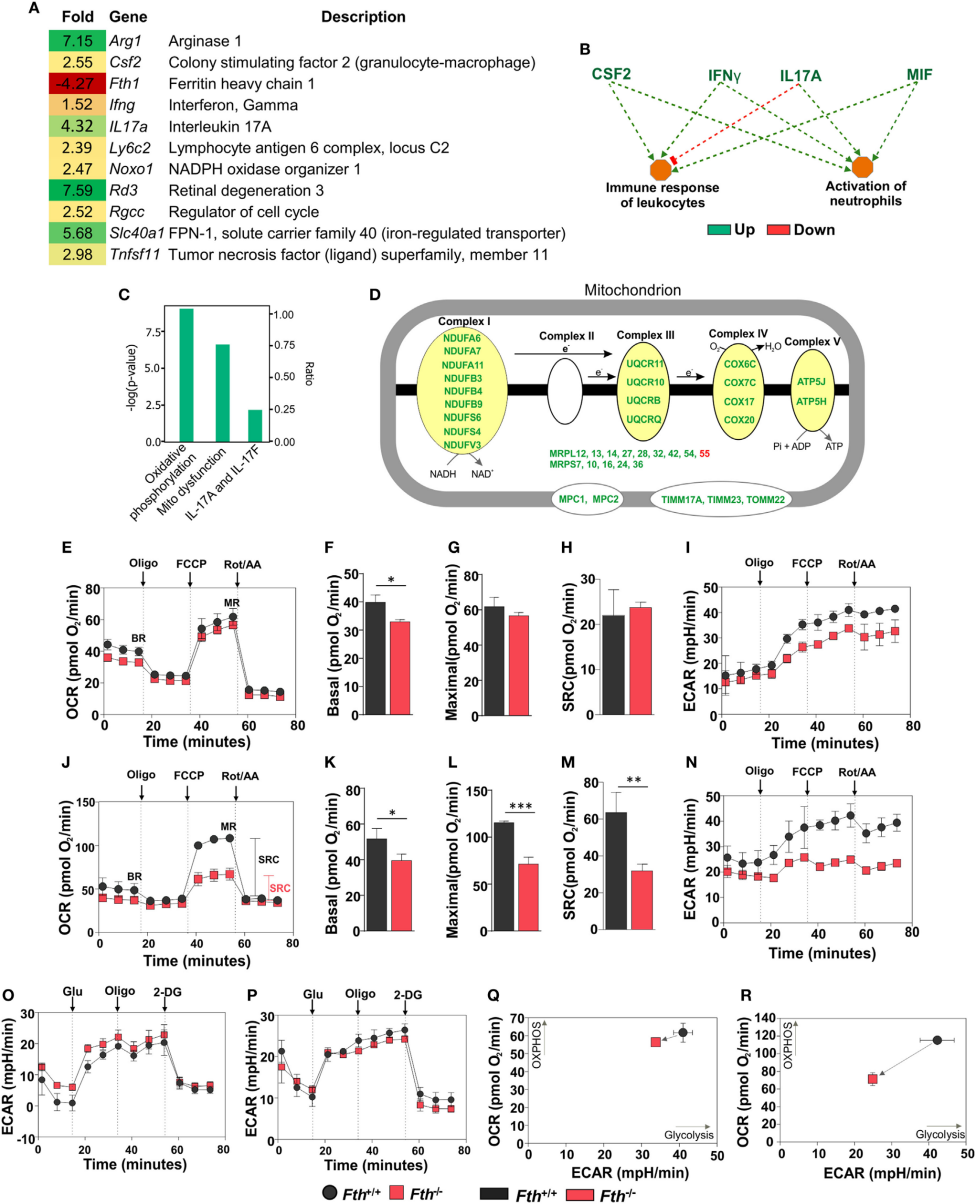
Gene expression analysis of lungs from *Mtb*-infected mice. **(A)** Heat map showing differentially regulated genes in the lungs of *Fth^−/−^* mice compared to wild-type mice at 9 weeks postinfection (*n* = 3). **(B)** Ingenuity pathway analysis (IPA) showing how upregulation of immunomodulators in *Fth^−/−^* mice affect immune functions. **(C)** IPA shows that pathways involved in oxidative phosphorylation, mitochondrial dysregulation, and the IL-17 family are altered in *Fth^−/−^* mice at 9 weeks postinfection. **(D)** IPA shows that pathways involved in the electron transport chain are altered in *Fth^−/−^* mice at 9 weeks postinfection. Bioenergetic analysis of peritoneal macrophages from *Fth^+/+^* and *Fth^−/−^* mice infected with *Mtb*. **(E)** Oxygen consumption rate (OCR) of uninfected peritoneal macrophages from *Fth^+/+^* and *Fth^−/−^* mice. **(F–H)** Basal respiration, maximal respiration (MR), and spare respiratory capacity (SRC) of uninfected macrophages. **(I)** Extracellular acidification rate (ECAR) of uninfected peritoneal macrophages from *Fth^+/+^* and *Fth^−/−^* mice. **(J)** OCR of *Mtb*-infected peritoneal macrophages at 24 h postinfection. **(K–M)** Basal respiration, MR, and SRC of *Mtb*-infected macrophages. **(N)** ECAR of *Mtb*-infected peritoneal macrophages at 24 h postinfection. **(O)** ECAR of uninfected macrophages using glycolytic stress test. **(P)** ECAR of *Mtb*-infected macrophages using glycolytic stress test. **(Q)** Phenogram (OCR Vs ECAR) of uninfected macrophages. **(R)** Phenogram (OCR Vs ECAR) of *Mtb*-infected macrophages. Data in panel **(A)** represents the mean fold change of three mice with *q* < 0.05 and cutoff ±1.5-fold. Statistical testing was performed using the unpaired Student’s *t*-test. Data are represented as mean ± SEM of three independent experiments (*p < 0.05, **p < 0.01, ***p < 0.001).

To identify the underlying mechanisms by which FtH contributes to protection against *Mtb* infection, we employed RNAseq to capture lung transcriptomes at 9 weeks post *Mtb* infection. We observed 72 differentially regulated genes in the lungs of *Fth^−/−^* mice compared to *Fth^+/+^* with a ≥2-fold change cutoff (Figure S4A in Supplementary Material). We observed a 4.27-fold reduction of FtH (*Fth1*) expression in the lungs of infected *Fth^−/−^* mice, consistent with the deletion of *Fth* in lung-residing myeloid cells (Figure 5A). Strikingly, expression of the iron exporter FPN (*Slc40a1*) was increased 5.68-fold in the lungs of *Fth^−/−^* mice, which is consistent with our immunoblot data (Figure 3A). We also observed a 7.15-fold upregulation of Arginase-1 (*Arg1*), a marker of M2 activation, which metabolizes Arg to polyamines such as putrescine and spermidine, which are essential for cell proliferation and tissue healing (18, 19, 42). Thus, *Arg1* upregulation may influence polyamine levels in *Fth^−/−^* mice. In addition, we observed a 7.59-fold increase in the expression of retinal degeneration 3 (*Rd3*) a gene associated with vision loss, in *Mtb*-infected *Fth^−/−^* mice (Figure 1D). Furthermore, genes involved in host immunity were upregulated in *Fth^−/−^* mice, including colony stimulating factor 2 (*Csf2*), and tumor necrosis factor superfamily, member 11 (*Tnfsf11*) and IFN-γ (*Ifng*) (Figure 5A), which is consistent with our cytokine data (Figures 4A–F). Finally, *IL17a* was also upregulated. This cytokine contributes to the production of pro-inflammatory cytokines and chemokines and is upregulated during *Mtb* infection (43).

Ingenuity pathway analysis (IPA) showed that upregulation of colony stimulating factor (*CSF2), IFN-*γ, IL-17A, and macrophage migration inhibitory factor (*MIF)* leads to activation of neutrophils and immune response to leukocytes (Figure 5B), and that ARG1, NOXO1, TNF SF11, and GM-CSF are involved in ROS synthesis (Figure S4B in Supplementary Material). Functional network analysis shows direct and indirect relationships between FtH, ARG1, FPN and other host proteins in lungs following *Mtb* infection (Figure S4C in Supplementary Material). Upregulation of FPN, which controls cellular iron release to the circulation, suggests that infected cells in the lungs of *Fth^−/−^* mice are attempting to maintain iron homeostasis *via* iron export.

### FtH Is Necessary for Maintaining Mitochondrial Oxidative Metabolism

Whereas most iron is stored in the cytosol by ferritin, the major flux of iron takes place in the mitochondrion during synthesis of Fe–S clusters and heme, which are key prosthetic groups utilized during OXPHOS. Since mitochondria control the metabolic programs in immune cells, which can be altered by pathogens to shift cellular differentiation and function (16, 44), we examined how *Mtb* exploits this metabolic vulnerability. IPA revealed that two pathways, “oxidative phosphorylation” and “mitochondrial dysfunction,” are significantly altered in the lungs of *Fth^−/−^* mice (Figure 5C). Indeed, we observed significant upregulation in the expression of a number mitochondrial respiratory genes associated with complexes I, III, IV, and V, as well as transport proteins (Figure 5D).

We next sought to test the hypothesis that FtH is necessary for maintaining bioenergetic homeostasis upon *Mtb* infection. An Agilent Seahorse XFp instrument was used to perform non-invasive, real-time measurement of the oxygen consumption rate (OCR), extracellular acidification rate (ECAR), spare respiratory capacity (SRC), and maximal respiration (MR) of *Mtb*-infected peritoneal macrophages. In this assay, a combination of inhibitors of respiratory complexes (oligomycin; Complex V, antimycin A, and rotenone; inhibit the ETC) and uncoupling agents [trifluorocarbonylcyanide phenylhydrazone (FCCP); stimulates MR] were used to examine and quantify different components of mitochondrial function. Using the mitochondrial stress test, we observed no significant differences in the OCR or SRC in uninfected peritoneal macrophages isolated from *Fth^−/−^* and *Fth^+/+^* mice (Figures 5E–H). ECAR profile of uninfected peritoneal macrophages from *Fth^+/+^* and *Fth^−/−^* mice is similar (Figure 5I). However, at 24 h postinfection, the OCR and SRC of *Fth^−/−^* macrophages were significantly reduced compared to *Fth^+/+^* cells (Figures 5J–M). The SRC is a measure of the capacity of a cell, when energy demand exceeded supply, to produce extra ATP and avoid an “ATP crisis.” Pharmacological or biological perturbation of MR, leading to a reduction of the SRC, is predictive of severe metabolic dysfunction. Thus, the reduction in SRC upon *Mtb* infection demonstrates that FtH deficiency severely compromises the ability of infected cells to rapidly generate energy upon demand. Consistent with these results, we also observed significantly reduced ECAR, an indirect measure of glycolysis, in *Fth^−/−^* peritoneal macrophages following *Mtb* infection (Figure 5N). We also measured the OCR of uninfected and *Mtb*-infected BMDM. Basal respiration in uninfected *Fth^+/+^* BMDM is slightly higher than *Fth^−/−^* BMDM, similar to our observations with peritoneal macrophages. We observed no differences in MR and SRC between uninfected BMDM from *Fth^+/+^* and *Fth^−/−^* mice (Figure S5A in Supplementary Material). Unlike peritoneal macrophages, *Mtb*-infected BMDM from *Fth^+/+^* and *Fth^−/−^* mice showed similar OCR profiles (Figure S5B in Supplementary Material).

We next performed a glycolysis stress test on peritoneal macrophages, which measures the ECAR. The ECAR measures proton extrusion resulting from glycolysis as well as the maximal glycolytic capacity of the cells. In this assay, infected cells are incubated in medium without glucose and the ECAR is measured. Following the addition of glucose, the ECAR is measured again during active glycolysis. To determine the maximum glycolytic capacity, an ATP synthase inhibitor is injected that shifts cellular energy production to glycolysis with a subsequent increase in ECAR. Finally, the glycolysis inhibitor 2-deoxy-glucose (2-DG) is injected, which confirms that the ECAR produced is due to glycolysis. Notably, we observed no difference in the ECAR between *Fth^−/−^* and *Fth^+/+^* macrophages before, or 24 h after infection (Figures 5O,P), suggesting that FtH does not play a role in glycolysis. By plotting OCR against ECAR, the difference in metabolic potential between uninfected (Figure 5Q) and infected (Figure 5R) *Fth^+/+^* and *Fth^−/−^* macrophages can be observed in the phenograms. To determine whether *Mtb* growth is altered in *Fth^−/−^* macrophages, we enumerated CFU from peritoneal macrophages and BMDM at 1, 3, and 5 days postinfection. In peritoneal macrophages, we observed slightly reduced growth of *Mtb* in *Fth^−/−^* cells compared to *Fth^+/+^* cells; however, there was no difference in the growth of *Mtb* recovered from *Fth^−/−^* and *Fth^+/+^* BMDM (Figures S6A,B in Supplementary Material).

In summary, these data demonstrate that a lack of FtH has little effect on the bioenergetics of uninfected macrophages. However, in infected peritoneal macrophages, the reduction in the SRC of *Fth^−/−^* macrophages provides strong evidence that FtH contributes to a distinct and critical component of bioenergetic homeostasis, OXPHOS, which is essential for protection against *Mtb* infection. These data are in full agreement with our RNAseq data (Figures 5A–D; Figure S4 in Supplementary Material), which also demonstrate mitochondrial dysfunction in *Fth^−/−^* macrophages following infection. Overall, this new evidence establishes a link between iron homeostasis and mitochondrial oxidative metabolism during *Mtb* infection.

### FtH Is Essential for Sustaining Energy Metabolism Upon *Mtb* Infection

**Figure 6:**
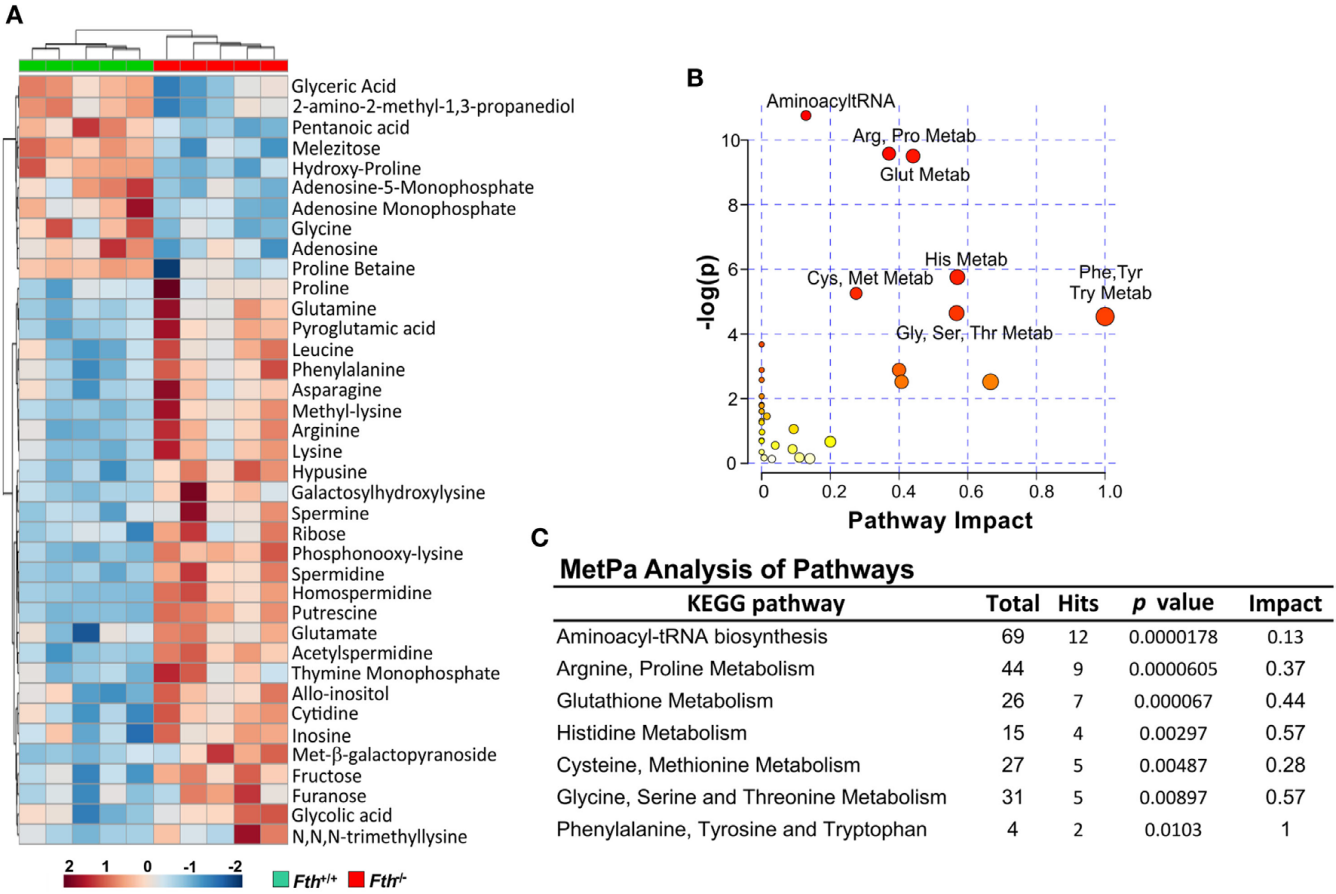
Metabolite clustering and MetPa Analysis of metabolites isolated from lungs of *Mtb-*infected mice. **(A)** Two-way hierarchical clustering analysis identified compounds measured by CE-MS and GC-MS of lung samples of *Fth^+/+^* and *Fth^−/−^* mice at 9-week postinfection. **(B,C)** MetPa analysis of metabolic pathways significantly altered in *Fth^−/−^* mice infected with *Mtb*. Statistical testing was performed using the unpaired Student’s *t*-test.

**Figure 7:**
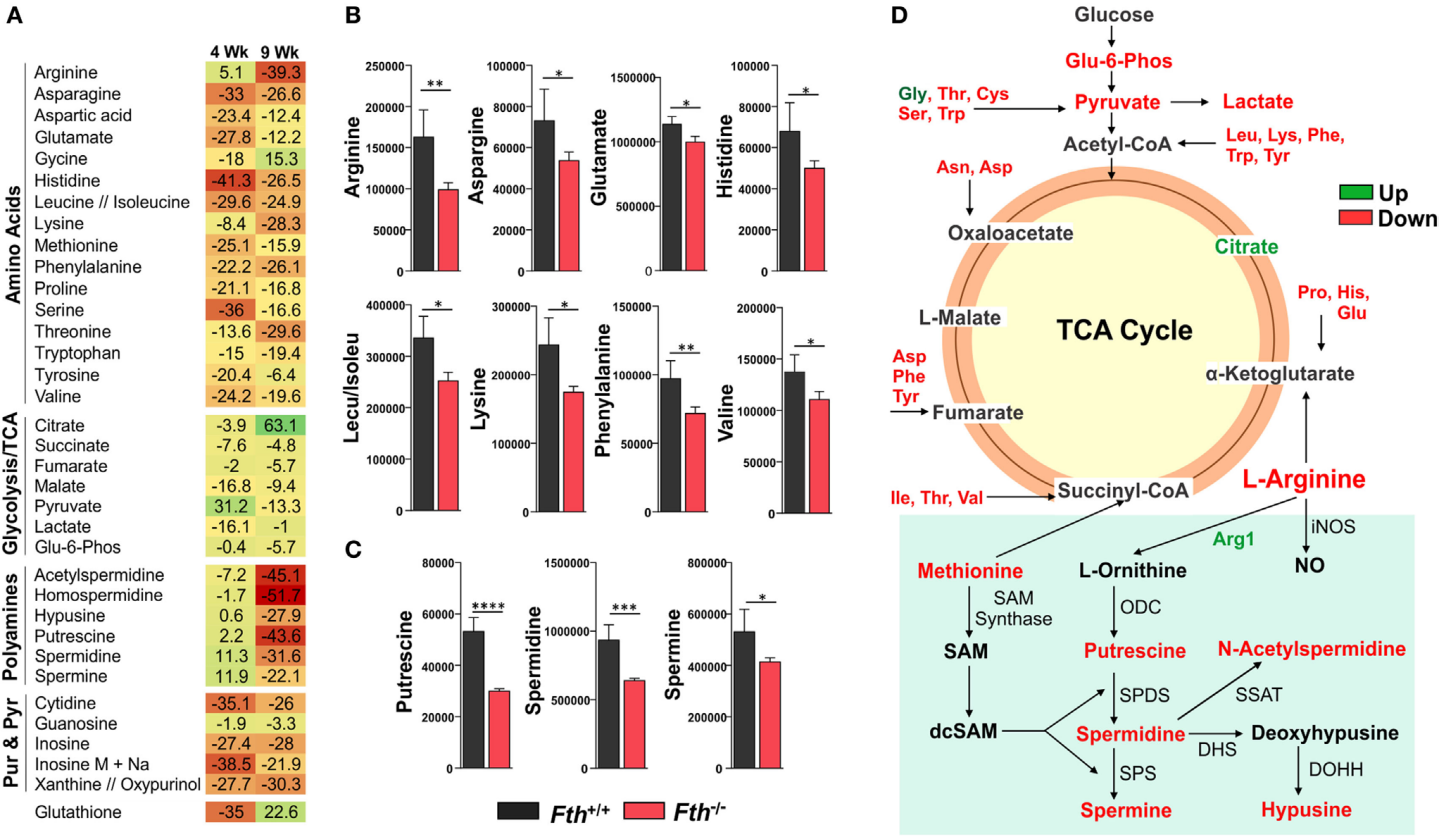
Metabolomic fingerprint in the lungs of *Mtb-*infected mice. **(A)** Heat map showing the percentage change in the levels of metabolites in the lungs of *Fth^−/−^* mice compared to *Fth^+/+^* mice at 4 and 9 weeks postinfection (*n* = 4). **(B)** Levels of essential amino acids in the lungs of mice at 4 weeks postinfection. **(C)** Levels of polyamines in the lungs of *Mtb*-infected mice at 4 weeks postinfection. **(D)** Amino acids feeding into the TCA cycle. Arg and polyamine biosynthesis pathway at 9 weeks postinfection in lungs of *Fth^−/−^* mice compared to *Fth^+/+^* mice. ODC, Ornithine decarboxylase; SPDS, spermidine synthase; SPS, spermine synthase; SSAT, spermidine and spermine acetyl transferase; DHS, deoxyhypusine synthase; DOHH, deoxyhypusine hydroxylase. Green text indicates increased levels of metabolite and red indicates reduced levels in the lungs of *Fth^−/−^* mice at 9 weeks postinfection. Statistical testing was performed using the unpaired Student’s *t*-test. Data are represented as mean ± SEM of four mice (*p < 0.05, **p < 0.01, ***p < 0.001, ****p < 0.0001).

Our bioenergetic data demonstrate that the metabolic demand of infected macrophages lacking FtH exceeds their supply, which is reflected by the cell’s reduced SRC. Identifying the mechanisms whereby FtH contributes to the SRC upon *Mtb* infection will provide insight into the role of iron in metabolic reprogramming. It is reasonable to assume that central carbon catabolism is directly involved as mitochondrial energy production is tightly linked to glycolysis, the pentose phosphate pathway, and the TCA cycle that feeds into the electron transport chain to produce ATP. Thus, we hypothesize that FtH is involved in reprogramming of central metabolism during *Mtb* infection. To test this hypothesis, we identified global metabolic changes associated with the lack of FtH during *Mtb* infection. We used a combination of capillary electrophoresis (CE-MS) and GC-MS to quantify metabolites in the lungs of *Mtb*-infected *Fth^+/+^* and *Fth^−/−^* mice. This dual platform approach is essential, as a single MS platform has been shown to be suboptimal (45). Two-way hierarchical clustering analysis of metabolites shows significant differences in the levels of several metabolites in the lungs of *Fth^+/+^* and *Fth^−/−^* mice at 9 weeks post *Mtb* infection (Figure 6A). MetaboAnalyst, which combines pathway enrichment analysis with pathway topology to detect metabolic differences, confirmed that the Arg and Pro, Phe, Tyr and Trp, and glutathione metabolic pathways are significantly altered in *Fth^−/−^* mice following *Mtb* infection (Figures 6B,C; Tables S1 and S2 in Supplementary Material). Notably, apart from Gly and citrate, all other energy metabolites were reduced in *Fth^−/−^* mice at 9 weeks postinfection (Figure 7A). Citrate levels were highly elevated, which is suggestive of a broken TCA cycle as this metabolite can feed into fatty acid synthesis and key macrophage effector molecules such as NO (17, 44). Of interest was the reduced levels of several essential amino acids and key glycolytic and TCA intermediates in *Fth^−/−^* mice (Figures 7A,B,D). In particular, Arg levels were the most depleted of all amino acids, and is consistent with our RNAseq data showing increased *Arg1* expression (Figure 5A). The fate of Arg in macrophages determines the inflammatory or anti-inflammatory cell phenotypes as iNOS can metabolize Arg into NO or catalyze polyamine production necessary for cell proliferation and tissue remodeling. Furthermore, Arg-derived Trp and Pro are mediators of the immunosuppressive activity of myeloid-derived suppressor cells (17, 19, 42), and tissue healing, respectively (19). Consistent with much reduced Arg, the polyamine metabolites putrescine, spermidine, spermine, hypusine, homospermidine, and acetylspermidine were also highly depleted in *Fth^−/−^* mice (Figures 7C,D).

In sum, these data establish a link between FtH-mediated iron homeostasis and core intermediary metabolism through anaplerotic and cataplerotic amino acid substrates, which converge to glycolysis and the TCA cycle to maintain energy metabolism. The broken TCA cycle together with a reduction in metabolites that participate in inflammation, suggest that lack of FtH exacerbates disease. Of particular significance is the *Arg*1/polyamine signature that links metabolism with immunity as polyamines are essential for regulating cellular proliferation, immunosuppression, and tissue restoration (16, 19, 46). Overall, the data established a critical link between FtH-mediated iron homeostasis and immune modulation.

### Iron Distribution in Human Tuberculous Lungs

Our mouse data point to an essential role for FtH-mediated iron homeostasis in the control of TB, which is evident from the lung iron distribution profile (Figures 2A,G,H). However, since mouse TB pathology does not fully reflect the spectrum of pathology in the human tuberculous lung (47–49), it is unclear whether species-specific pathophysiological differences have any effect on iron distribution. Thus, it is difficult to accurately assess the clinical relevance of iron distribution and deposition in mice. Notably, we were unable to identify published studies that examine iron deposition directly in human TB lung tissue. Thus, to assess the clinical relevance of our mouse studies and whether heterogeneity in tuberculous lesions contributes to iron deposition, we examined iron distribution in archived sections from human TB lungs, and determined the iron concentration in freshly resected lungs of TB patients and healthy controls.

**Figure 8:**
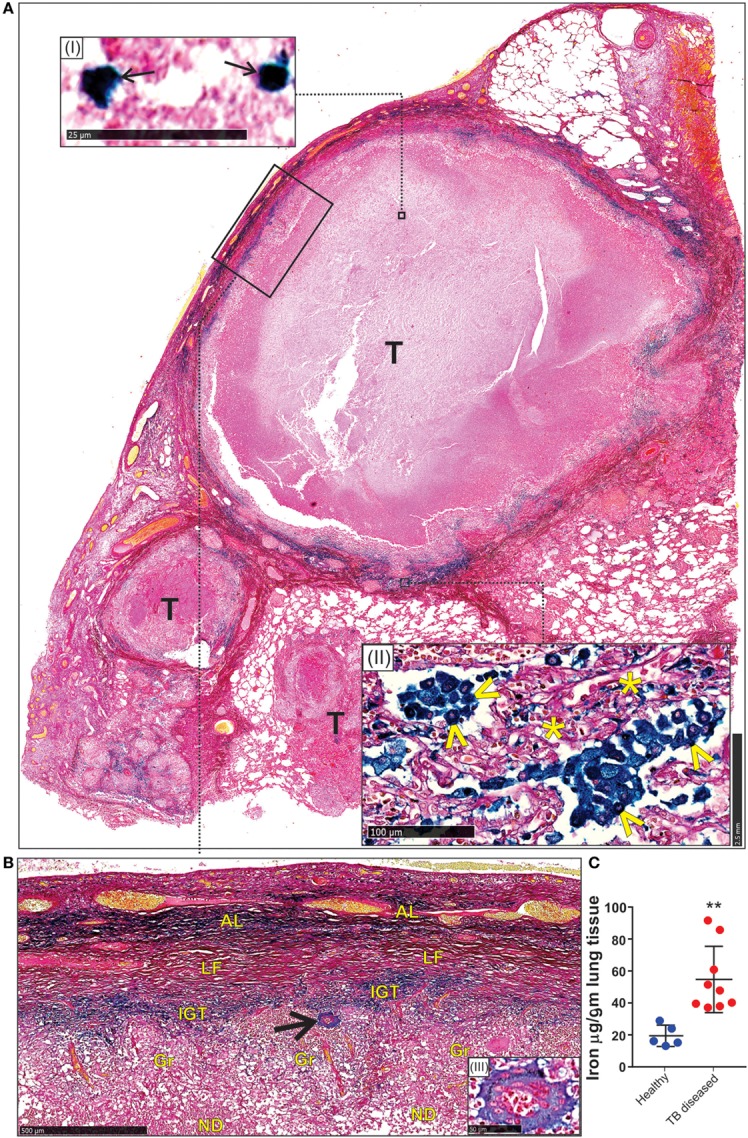
Spatial distribution of iron in the human lung tubercle. **(A)** Low power depiction of Iron van Gieson (FeVG stains hemosiderin a sea-green, and collagen a red color) stained lung section highlighting tubercles (T). Inset (I): high power demonstration of scattered sea-green siderophages (arrows) in central necrotic caseative debris. Inset (II): abundant hemosiderin in adjacent lung tissue within patent (arrowheads) and collapsed (asterisks) alveolar spaces. **(B)** High power depiction of hemosiderin distribution in fibro-inflammatory response in rectangle **(A)** with pale hemosiderin in focal histiocytes and giant cells [arrow and inset (III)] within granulomas (Gr). Conspicuous iron in inflamed granulation tissue (IGT). Less, but noticeable iron in outer lamellar fibrosis (LF) (AL, adjacent lung; ND, necrotic debris in caseative focus). **(C)**. Iron levels in the lungs of healthy and TB patients (**p = 0.0034).

**Figure 9:**
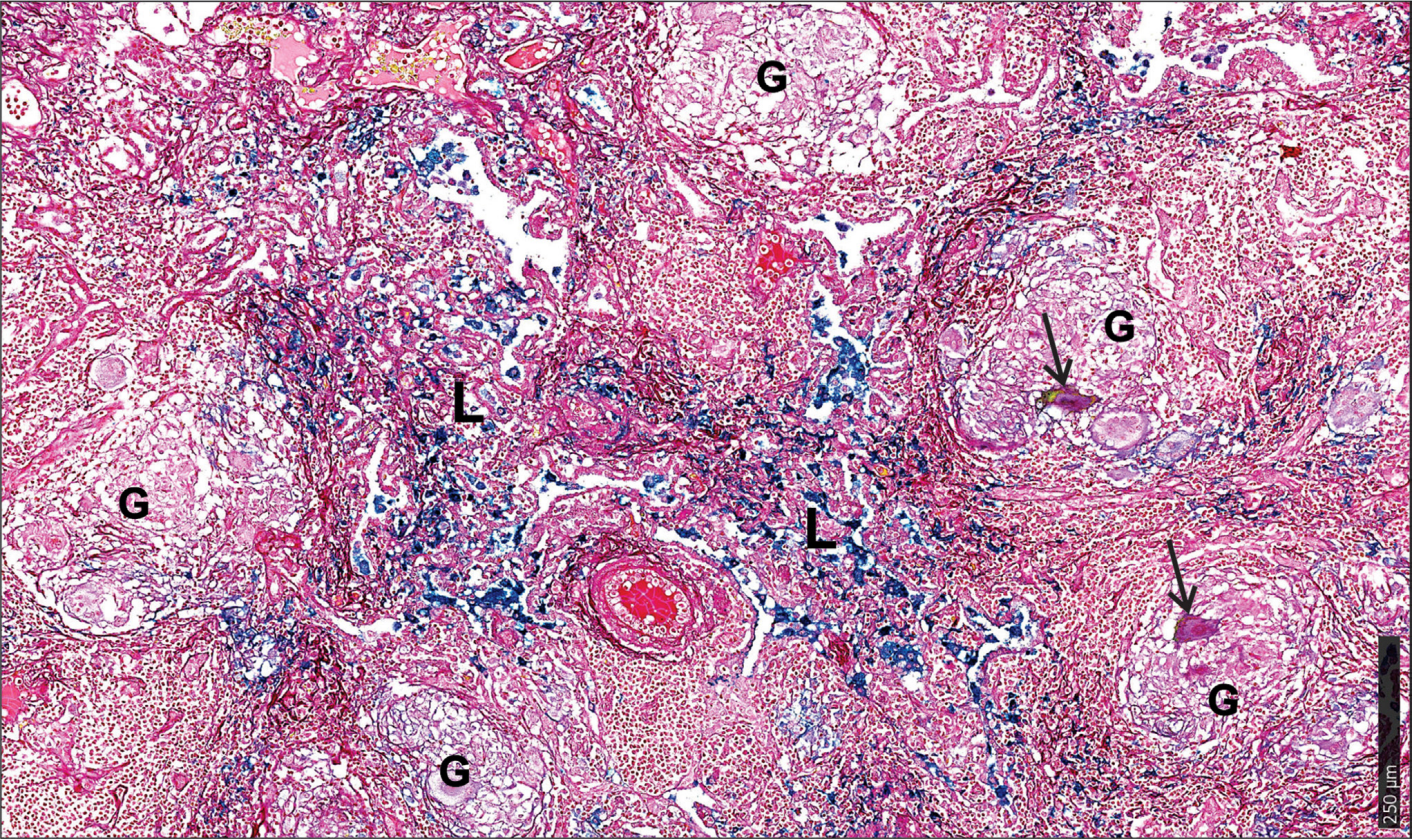
Microscopic iron distribution in the human tuberculous lung. FeVG-stained section of non-necrotizing granulomatous inflammation (NNGI) showing confluent, NNGI with stippled pale staining hemosiderin in organized granulomas (G) and in Langhans giant cells (arrows). Conspicuous sea-green hemosiderin deposits in intervening and surrounding, variably fibrotic, lung (L) parenchyma.

First, we used the iron van Gieson (FeVG) stain that employs the Prussian blue staining reaction, which stains insoluble, metabolically inert iron–ferritin complexes, also referred to as hemosiderin (50–52). It should be noted that iron deposition in the lungs as measured by FeVG staining is a compelling pathological correlate in patients suffering from pulmonary diseases (53–56). Hemosiderin was noted very focally in caseative debris (Figure 8A, inset i). Hemosiderin was prominent in the inflammatory granulation tissue that surrounded the granuloma and stained brightly and prominently in patients with collapsed resident alveolar spaces (Figure 8A, inset ii) Pale hemosiderin staining was present in the granulomas and hemosiderin aggregation in the cytoplasm of a giant cell (Figure 8B, inset iii). Interstitial fibrosis was noted in close association with hemosiderin. In addition, scattered and confluent non-necrotizing granulomas (NNG) were also identified on microscopic examination (Figure 9). In these foci, hemosiderin deposition was most prominent in the intervening and surrounding patent and collapsed resident alveolar parenchyma (Figure 9). There were multiple foci of fresh hemorrhage with conspicuous erythrocyte extravasation and congestion (Figures 8 and 9). Sections of normal lung tissue did not contain tuberculous lesions or hemorrhage on routine staining, and no hemosiderin deposits were observed on FeVG staining.

Second, we determined the iron levels in the lungs of nine active TB patients and five healthy controls. Resected *Mtb*-infected human lung tissue was obtained following surgery for removal of irreversibly damage lobes or lungs, and provided us with a unique opportunity to examine how *Mtb* contributes to iron dysregulation. Our data indicate that the lungs of *Mtb*-infected patients contain significantly more iron (54.7 ± 6.9 µg/g tissue; *n* = 9) than healthy controls (19.4 ± 2.9 µg/g tissue; *n* = 5) (Figure 8C).

In sum, the enhanced iron levels and distinct iron distribution pattern in human tuberculous lungs reflect the destructive tuberculous lesions that damage lung parenchyma and resident pulmonary vessels, with subsequent intrapulmonary hemorrhage and tracking of blood through bronchial and bronchiolar airways and intra-alveolar spaces. In contrast, while the iron distribution pattern in the murine model reflected the profile of perivascular and peribronchial hemorrhage, the simple distribution pattern in the murine model reflected the relatively simple underlying histiocytic aggregation (Figures 2G,H) rather than tubercle formation, confluent organized, NNG, and interstitial fibrosis in human tissue (Figures 8 and 9). These findings provide conclusive evidence that *Mtb* infection of the human lung severely disrupts iron homeostasis in distinct microanatomic locations and contributes to lethal immunopathology.

## Discussion

While it is widely acknowledged that *Mtb* requires host iron to survive and cause disease, the underlying mechanisms by which the host controls iron availability during infection remain unresolved. Here, we provide evidence that the iron storage protein FtH plays a critical role in protecting mice against *Mtb*. The mechanism of this protection includes maintenance of mitochondrial function and shifting central intermediary metabolism, which help control the fate of immune cells. FtH deficiency leads to metabolic dysregulation as shown by reduction of mitochondrial SRC, depletion of TCA and glycolytic intermediates and several essential amino acids, and impairment of the ARG1–polyamine axis. Unexpectedly, lack of FtH triggers iron export through FPN during infection, which limits iron availability for *Mtb* growth and disease. Distinct iron deposition and distribution profiles, as well as iron concentrations in human tuberculous inflammatory granulation lesions and tissue, respectively, established that *Mtb* contributes to iron dysregulation and subsequent pathology. Hemorrhage was identified as a major cause of iron dysregulation in human tuberculous lung. Establishing the mechanisms whereby FtH mitigates metabolic vulnerability during infection represents a critical advancement of our understanding of iron metabolism in TB. We anticipate our findings will establish a new paradigm for how iron homeostasis contributes to energy metabolism for protection against TB.

Iron is a cofactor for heme, iron–sulfur cluster, and oxygen-dependent proteins in mitochondrial respiration, a process essential for energy production. In this study, an important contribution was establishing a role for FtH in meeting the metabolic and bioenergetic needs of cells following *Mtb* infection. Several independent lines of evidence support this conclusion. First, RNAseq analysis revealed the differential expression of several genes involved in mitochondrial transport and ETC complexes I, III, IV, and V in infected *Fth^−/−^* mice, suggesting bioenergetic dysregulation. Second, we confirmed mitochondrial dysfunction upon *Mtb* infection using extracellular metabolic flux analysis. This highly sensitive technique allowed us to noninvasively quantify the contribution of FtH to the bioenergetics of infected host cells in real time. An important bioenergetic parameter, the SRC, was significantly reduced in *Fth^−/−^* cells by *Mtb*. The SRC is critical for maintaining cellular function during acute or chronic stress conditions, and is an indicator of the cell’s ability to produce extra ATP during increased energy demand (57). Consequently, reduced SRC is associated with disease and cell death. Recent studies have pointed to pyruvate and Complex II (succinate dehydrogenase) as potential sources of the SRC (58, 59). Consistent with these studies, we observed substantially reduced levels of succinate and pyruvate during *Mtb* infection. An important observation was that the bioenergetic capacity in uninfected *Fth^−/−^* and *Fth*^+/+^ peritoneal macrophages was virtually identical, whereas in infected *Fth^−/−^* peritoneal macrophages the SRC was reduced. This suggests that FtH deficiency exerts a distinct effect on host bioenergetics following infection that is reflected only by the SRC, and not a general response. Distinct from our peritoneal bioenergetic data, we observed similar OCR profiles in infected *Fth^−/−^* and *Fth*^+/+^ BMDM. These data, as well as our peritoneal and BMDM CFU data (Figures S6A,B in Supplementary Material) suggest that macrophages from anatomically distinct locations and developmental origins have discrete bioenergetic capacities, which respond differently during *Mtb* infection (60).

Third, our metabolomics results, generated from two independent MS platforms, supported our real-time bioenergetic findings and showed that several essential amino acids, TCA and glycolytic intermediates are significantly depleted in lungs of *Mtb*-infected *Fth^−/−^* mice. Notably, Arg was the most depleted, which is consistent with our RNAseq data showing upregulation of *Arg1* in the lungs of *Fth^−/−^* mice. Arg plays an important role in the inflammatory function of macrophages and is a substrate for iNOS to produce NO and (in the arginase pathway) to produce polyamines, glutamate, and proline (16, 19). Intriguingly, we observed substantially reduced levels of the polyamine inter-mediates acetylspermidine, homospermidine, hypusine, putrescine, spermidine, and spermine, as well as Glu and Pro in *Fth^−/−^* mouse lungs. The synthesis of polyamines is downstream from Arg metabolism and they are essential for cell growth, proliferation, and tissue healing under pathophysiological conditions (19, 42, 61). We, therefore, posit that reduced levels of polyamines in *Fth^−/−^* mice diminish tissue modeling, repair, and cell proliferation to exacerbate TB disease. Further, Arg is a precursor of Pro, the building block of collagen (19), and Glu, which also regulates numerous aspects of immune cell function including NO and IL-1 (17) and, therefore, emphasizes the crucial role this amino acid play in FtH-mediated iron dysregulation. On the other hand, polyamines can also chelate iron during iron overload and act as non-transferrin bound iron components to control intracellular iron levels (62). Arg depletion could also cause a reduction in NO levels to modulate the host inflammatory response as was shown for *Leishmania* (63). However, since *Arg1*-deficient mice are resistant to *Mtb* infection (64) it suggests that multiple factors are involved in case of TB.

Elegant studies have shown that the different functional populations of immune cells use distinct metabolic programs which, if altered, can shift cellular function (15). As a result, while T cells and macrophages have substantial plasticity to obtain energy for survival and proliferation, changes in environmental substrates can alter or impair immune function (15, 44). Our metabolomic data demonstrate that during acute and chronic infection there is depletion of metabolic substrates in the lungs, which likely alters immune function. Adequate levels of amino acids such as Leu, which is significantly depleted in *Fth^−/−^* mice, are needed to preserve mTOR-driven anabolic growth and immune function (65, 66). Although it could be argued that the increased *Mtb* burden metabolizes amino acids as reported for *Listeria monocytogenes* (67), a likely alternative is that some amino acids are preferentially metabolized (or not) by certain immune cell populations. For example, Glu *via* Gln can be used as an alternative substrate for the TCA cycle to support ATP production, or to support fatty acid synthesis *via* citrate whereas Trp strongly supports cellular proliferation and anabolic growth (17, 68). Glu and Trp are noticeably depleted in *Fth^−/−^* mice (Figure 7D). Finally, Ser, Arg, and Cys are essential for T-cell proliferation and function (69–72), illustrating the importance of amino acids in regulating adaptive immune responses. Hence, the reduced levels of some of these amino acids in the lungs of infected *Fth^−/−^* mice may compromise immune function leading to a susceptible phenotype.

Since metabolism is intimately linked to immune cell function and fate (15, 16, 44), it is not surprising that the dysregulated metabolism in *Fth^−/−^* mice is skewed toward excessive inflammation. This was evident from our RNAseq data showing upregulation of genes involved in inflammation (e.g., *Arg1, Ifng, Tnfsf11, IL17a*), IPA-based identification of several inflammatory pathways (e.g., “activation of neutrophils,” “immune responses of leukocytes,” etc.) and upregulation of IFN-γ, TNF-α, IL-8, IL-2, and GM-CSF. This was further confirmed by flow cytometry data showing substantial increase in neutrophils in infected *Fth^−/−^* mice. Therefore, it is reasonable to conclude that this excessive inflammatory response contributes to the markedly reduced survival of *Fth^−/−^* mice, particularly as IFN-γ and TNF-α are associated with the control of iron homeostasis (73, 74).

Iron homeostasis in the host circulation is maintained by iron absorption in the duodenum, recycling of iron from erythrocytes and mobilization from iron stores in host cells. These events are tightly controlled by the FPN/HEP axis (23). HEP binds to FPN and induces its internalization, thereby blocking iron release into the circulation (24, 75). *Mtb* infection leads to significantly reduced levels of iron in the lungs and spleens of *Fth^−/−^* mice and is a likely defense strategy to reduce iron availability to *Mtb*. This is supported by increased production of lung FPN (Figures 3A and 5A), which exports iron from organs into the circulation based on host requirements (9, 22). *Fth^−/−^* mice exhibited markedly increased HEP levels in the serum compared to *Fth^+/+^* mice at 4 and 9 weeks postinfection. Consistent with increased HEP levels, we observed significantly reduced levels of FPN in the duodenum of infected mice. With limiting FPN in the duodenum, iron is trapped in the duodenal enterocytes and ultimately shed into the gut, resulting in increased iron in the feces, as we observed (Figure 2F), and as was reported previously (76). Of note, we were unable to identify from the literature any other microbial pathogen that triggers a reduction of FPN in the duodenum leading to increased iron levels in the feces.

Our pathology and iron quantitation data agree with each other and provide compelling evidence for dysregulated iron homeostasis in the pathophysiology of human TB. Surprisingly, we were unable to identify published studies that examine iron deposition or iron content directly in human TB lung tissue. It should be emphasized that excessive iron deposition in the lungs is an important pathological correlate in patients suffering from pulmonary diseases, including diffuse alveolar hemorrhage and idiopathic pulmonary fibrosis, and is not observed in healthy lung tissue (54–56, 77). Detection of hemosiderin mainly in lung parenchyma indicates that the predominant source, hemorrhage, is external to the tuberculous lesions. Since hemosiderin represents the product of the breakdown of hemoglobin released from erythrocytes in hemorrhagic foci, our data suggest that hemorrhagic inflammation resulting from ruptured alveolar walls and the release of large quantities of heme-derived iron is the prominent source of iron deposition. In tubercles, hemosiderin was seen predominantly within the inflamed granulation tissue layer, a consequence of hemorrhage from the leaky, neovascularized compartment (Figure 8A). The minimal hemosiderin within the granulomas and giant cells in necrotic and non-necrotic granulomatous is likely a function of the phagocytic capacity of the giant cells and histiocytes, albeit of weak potential.

**Figure 10:**
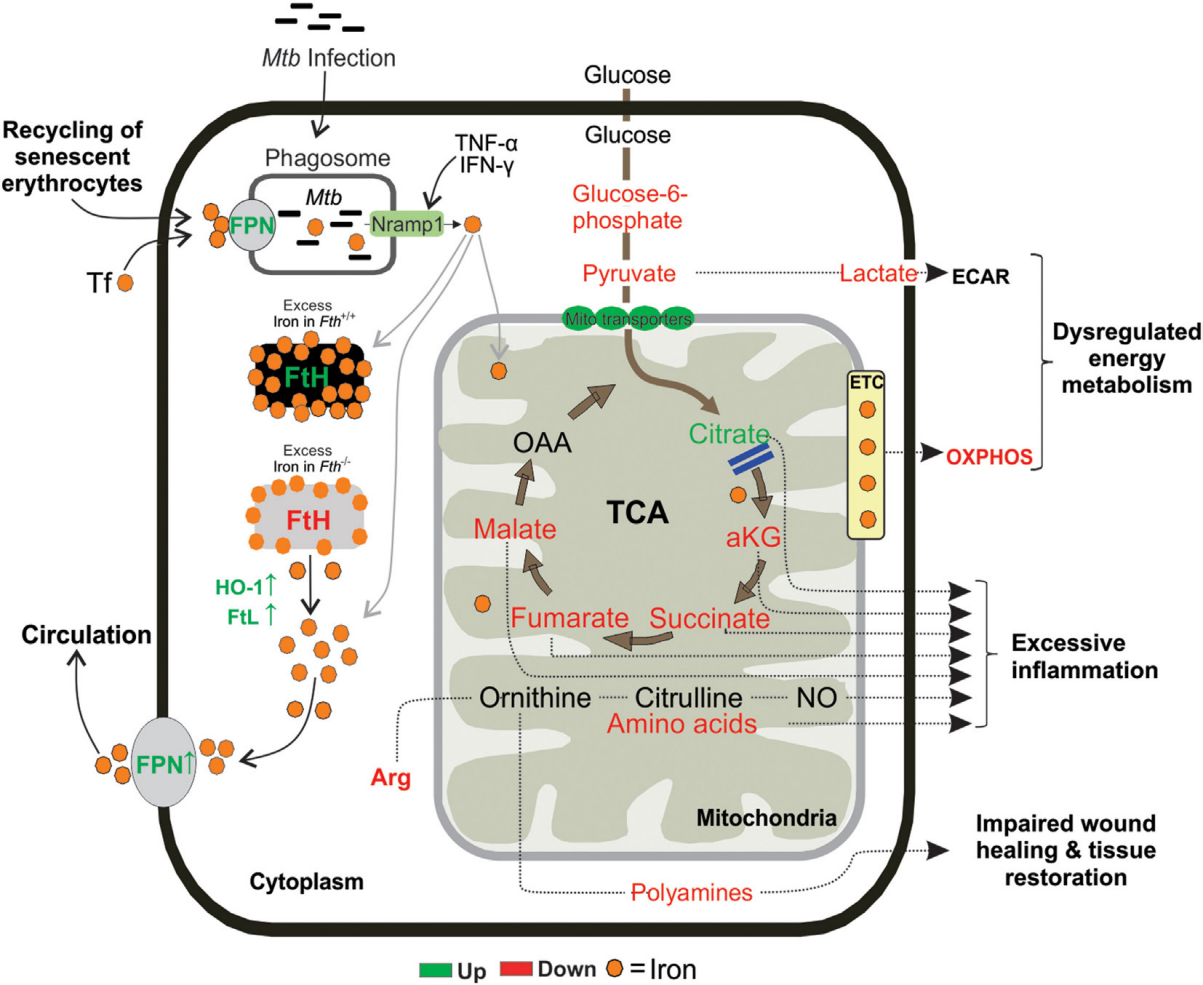
Proposed mouse model. Following exposure, *Mtb* resides inside the lung macrophages and replicate by using host iron to cause infection. *Fth^−/−^* mice tries to reduce the availability of iron in the lung cells by upregulating FPN, an iron exporter. FtH is essential for maintaining mitochondrial function through oxidative phosphorylation (OXPHOS) and demonstrated an essential role for FtH in maintenance of central intermediary metabolism.

In conclusion, in the murine model of TB, we have identified unsuspected mechanisms whereby *Mtb* exploits the metabolic vulnerability of dysregulated iron metabolism. Our data suggest that FtH-mediated iron dysregulation interrupts metabolic pathways in the lungs of infected mice to shift immune cell fate to excessive inflammation, extensive dissemination of *Mtb*, and lethal pathology. Our data show that FtH deficiency links central carbon metabolism and mitochondrial oxidative metabolism to the inflammatory response (Figure 10). We provide *in vivo* evidence that *Mtb* infection critically affects iron absorption and impairs the key regulator of iron homeostasis, the FPN/HEP axis. We also show that in human TB the presence and distribution of iron was inevitably associated with destructive tuberculous lesions and hemorrhage. Our study raises the possibility that pharmacological reprogramming of host energy metabolism (78), iron homeostasis through FPN/HEP (25) or anti-fibrogenic agents could be used to prevent either iron overload or reduce iron levels to improve the health of TB patients.

## Materials and Methods

### *Mtb* Culture Conditions

*Mycobacterium tuberculosis* H37Rv was grown at 37°C with shaking in BD Difco Middlebrook (MB) 7H9 (broth) or standing on MB7H11 (agar) media supplemented with 0.2% glycerol, ADS (Albumin, Dextrose, NaCl) with 0.02% tyloxapol.

### Mice

C57BL/6 *LyzM^cre^Fth*^Δ^*^/^*^Δ^ (*Fth^−/−^*) mice were generated by crossing C57BL/6 *Fth^lox/lox^* mice (79) (provided originally by Lukas Khun, ETH, Switzerland) with C57BL/6 *LyzM^Cre^* (80) (Jackson Laboratories; stock 004781). Mice were housed in a pathogen-free facility and were used according to procedures approved by the IACUC at the University of Alabama at Birmingham. Mice genotype was confirmed by PCR and western blotting. Both male and female mice (10–12 weeks) were used in this study.

### *Mtb* Infection

For survival experiments, mice were infected with a high dose (5 × 10^4^) and a low dose (1 × 10^4^) of *Mtb* H37Rv *via* the intratracheal route. Bacterial numbers were determined by plating homogenized organs on MB 7H11 agar plates. For *Fth^−/−^* mice, the day 1 CFU for the high dose was 2,710 and 2,630, respectively, in two animals, and the medium dose was 310 and 330, respectively, in two animals. For *Fth^+/+^* mice, the day 1 CFU for the high dose was 2,200 and 2,740, respectively, in two animals, and the medium dose was 270 and 350, respectively, in two animals. For the time point experiment, mice were infected with 5 × 10^4^ *Mtb* H37Rv *via* the intratracheal route. Four and nine weeks postinfection, the lungs, spleen, brain, and eyes were harvested, homogenized in PBS pH 7.4 and serial dilutions of homogenates were plated onto Middlebrook (MB) 7H11 agar containing carbencillin (25 µg/ml) and cycloheximide (25 µg/ml) to determine bacillary count.

### Isolation of BAL Fluid From Mice and Cytokine Analysis

After euthanasia and exposure of the trachea, a Safelet Cath (22 g × 1″, Exel) was inserted into trachea and BAL was performed using 1 ml PBS pH 7.4. The lavage sample was centrifuged at 2,500 × *g* for 5 min at 4°C to pellet the cells and the supernatant was used for cytokine analysis. Both Th-1 and Th-2 cytokines were analyzed using a Bio-Plex Pro™ Mouse Cytokine Th1/Th2 Assay and Bio-Plex Pro™ Mouse Cytokine 23-plex Assay (Bio-Rad). IL-8 was quantified in BAL fluid using the Mouse IL-8 ELISA kit (MyBioSource) according to the manufacturer’s instructions. Briefly, a 48-well plate was coated with the primary capture Ab and stored overnight at 4°C. Following washes with PBS-T, the plate was blocked with assay diluent for 1 h at room temperature. Detection Ab and diluted Avidin-HRP were then added to the plate, with washes between additions, and the plate was developed with TMB substrate in the dark. Absorbance was then measured at 450 nm.

### Flow Cytometry

Single cell suspensions from lungs and spleens were prepared by chopping tissue into small pieces followed by digestion with Liberase DL (Roche) at 37°C for 30 min. RBC were removed by treatment with ACK lysis buffer followed by washing with PBS pH 7.4. Cells were treated with fixable viable dye (eBiosciences) for 30 min at 4°C in the dark. Fc receptors were blocked with anti-CD16/32 for 10 min at 4°C in the dark. Finally, cells were resuspended in staining buffer (PBS pH 7.4 containing 0.1% BSA and 0.01% NaN_3_) and stained at saturating concentrations of antibodies specific for CD11b, #83-0112; CD45, #86-0451; Ly-6C, #48-5932; Ly-6G (GR-1), #17-9668; and CD4, #83-0042 (eBiosciences). Samples were fixed in 2% paraformaldehyde solution for 20 min and data collected using an LSRII flow cytometer (Becton-Dickenson) and analyzed using Winlist Software (Verity Software House, Inc.), FlowJo Software (Tree Star, Inc.).

### Evans Blue Invasion

Evans blue solution (0.5%) was prepared in PBS pH 7.4 and sterile filtered. Mice were infected with *Mtb* and 4 weeks postinfection mice received an i.v. injection of 200 µl of 0.5% Evans blue solution. Mice were sacrificed 30 min after Evans blue injection and the brain was visually examined for the dye. Evans blue dye was quantified (absorbance at 600 nm) following extraction from brain tissue using a formamide solution at 56°C for 2 days as described previously (81).

### Inductively Coupled Plasma Mass Spectrometry (ICPMS)

Total iron in the lungs, spleen, and serum was quantified by ICPMS at Mass Spectrometry Research Center, Vanderbilt University, USA. Briefly, samples (~100 mg) were mineralized in teflon vials with pure nitric acid (67–69%) and hydrogen peroxide (36%) at 90°C for 16 h. Iron quantification of diluted acid digested samples was performed on the Element 2 ICPMS (Thermo Fisher Scientific, Bremen, Germany) coupled with ESI auto sampler (Elemental Scientific, Omaha, NE, USA). The sample uptake was achieved through self-aspiration *via* 0.5 mm ID sample probe and sample capillary. Iron was measured based on isotope ^56^Fe at medium resolution (*R* = 4,300).

### Iron Quantitation in Mice

Iron levels in urine and feces were measured using the Iron Assay Kit (Abcam) according to the manufacturer’s recommendations. Iron levels in the urine were normalized to urine creatinine levels (UAB O’Brien Center for Acute Kidney Injury Research).

### Serum Hepcidin Quantitation

Blood was collected from uninfected and *Mtb*-infected mice and serum was isolated. Serum hepcidin levels were measured using a Mouse Hepc (Hepcidin) ELISA Kit (Elabscience Biotechnology Co., Ltd., Wuhan, China) according to the manufacturer’s instructions.

### Western Blotting

*Mtb*-infected mouse lungs were lysed in RIPA buffer containing protease inhibitors (cOmplete Tablets, Roche) using a dounce homogenizer. Protein was quantified using the BCA protein assay kit (Thermo Scientific). 20 µg of total protein was resolved on a 4–15% gradient SDS-PAGE gel (Bio-Rad) and transferred to a PVDF membrane. Membranes were blocked in 5% nonfat dry milk in PBS-T for 1 h and then incubated with a primary antibody followed by a peroxidase-conjugated secondary antibody. HRP activity was detected using ECL Western Blotting reagent (Amersham).

### Antibodies

Primary antibodies used in this study are Ferritin heavy chain, H-53, #sc-25617, Santa Cruz Biotechnology (1:5,000); Ferritin light chain, D-18, #sc-14420, Santa Cruz Biotechnology (1:5,000); HO-1 antibody, #ADI-SPA-895, Enzo life sciences (1:5,000); FPN/SLC40A1, #ab85370, abcam (1:2,500); GAPDH, FL-335, sc-25778, Santa Cruz Biotechnology (1:5,000) and secondary antibodies used in this study are Goat anti-Rabbit IgG HRP #ab6721, Abcam (1:20,000), Donkey anti-goat IgG HRP, #sc2020, Santa Cruz Biotechnology (1:20,000), Rabbit Anti-Mouse IgG H&L HRP, #ab6728, Abcam (1:20,000).

### RNA Extraction From Lung Tissues

Total RNA from lungs of *Mtb*-infected *Fth^+/+^* and *Fth^−/−^* mice was extracted using RNeasy Plus mini kits as per manufacturer’s recommendations (Qiagen).

### Next-Generation Sequencing on Illumina Platforms

Sequencing of mRNA was performed on the Illumina HiSeq2500. Briefly, the quality of the total RNA was assessed using the Agilent 2100 Bioanalyzer followed by two rounds of poly A+ selection and conversion to cDNA. A TruSeq library generation kit was used as per the manufacturer’s instructions (Illumina, San Diego, CA, USA). Library construction consisted of random fragmentation of the polyA mRNA, followed by cDNA production using random primers. The ends of the cDNA were repaired, A-tailed and adaptors ligated for indexing (up to 12 different barcodes per lane) during the sequencing runs. The cDNA libraries were quantitated using qPCR in a Roche Light Cycler 480 with the Kapa Biosystems kit for library quantitation (Kapa Biosystems, Woburn, MA, USA) prior to cluster generation. Clusters were generated to yield approximately 725K–825K clusters/mm^2^. Cluster density and quality were determined during the run after the first base addition parameters were assessed. Performed paired end 2 × 50 bp sequencing runs to align the cDNA sequences to the reference genome.

TopHat was used to align the raw RNA-Seq fastq reads to the mouse mm10 genome using the short-read aligner Bowtie (82, 83). TopHat also analyzes the mapping results to identify splice junctions between exons. Cufflinks uses the aligned reads from TopHat to assemble transcripts, estimate their abundances, and test for differential expression and regulation (84). Cuffmerge merges the assembled transcripts to a reference annotation and is capable of tracking Cufflinks transcripts across multiple experiments. Finally, Cuffdiff finds significant changes in transcript expression, splicing, and promoter use. Genes that meet desired criteria (i.e., fold change ≥ ±2.0 and *q* value < 0.05) were further analyzed using Ingenuity’s Pathway Analysis tool.

### Isolation of Macrophages

Peritoneal macrophages: peritoneal macrophages were isolated from *Fth^+/+^* and *Fth^−/−^* mice using aseptic technique. RBCs were removed by lysis with ACK lysis buffer followed by washing with PBS pH 7.4. Peritoneal macrophages were infected with *Mtb* and CFU determination and extracellular flux analysis were performed. BMDM: *Fth^+/+^* and *Fth^−/−^* mice were sacrificed, and femurs and tibia were removed using aseptic technique. BMDM were isolated by flushing the femurs with PBS pH 7.4. RBCs were removed by lysis with ACK lysis buffer followed by washing with PBS pH 7.4. Protein was isolated from BMDM and western blot analysis was performed to evaluate FtH and FtL expression. BMDM were infected with *Mtb* and CFU determination and extracellular flux analysis were performed.

### Mitochondrial Stress Test

Peritoneal macrophages and BMDM were isolated from uninfected mice. Macrophages were infected with *Mtb* at an MOI of 1:1 (macrophage:*Mtb*). 24 h after infection, mitochondrial respiration was measured in macrophages as the OCR using the Mito Stress Test kit (Seahorse Biosciences) according to the manufactures recommendations. Three OCR measurements were made after the sequential addition of Oligomycin (1.5 µM), FCCP (1.5 µM), and Rot/AA (0.5 µM) through the drug ports of the sensor cartridge. Each test was performed in triplicate using 2 × 10^5^ cells/well in an XFp flux analyzer and the data were analyzed using Wave Desktop 2.2 software.

### Glycolysis Stress Test

Peritoneal macrophages were infected with *Mtb* at an MOI of 1:1 (macrophage:*Mtb*). 24 h after infection, glycolysis was measured in macrophages as the ECAR using the Glycolysis Stress Test kit (Seahorse Biosciences) according to the manufacturer’s recommendations. Three ECAR measurements were made after the sequential addition of Glucose (10 mM), Oligomycin (1.5 µM), and 2-DG (50 mM) through the drug ports of the sensor cartridge. Each test was performed in triplicate using 2 × 10^5^ cells/well in an XFp flux analyzer and, the data were analyzed using Wave Desktop 2.2 software.

### *Mtb* Infection of Macrophages

Peritoneal macrophages and BMDM were infected with *Mtb* at an MOI of 1:3 (macrophage:*Mtb*), and the number of intracellular viable bacteria was determined at 4 h (Day 0), 1, 3, and 5 days postinfection. Cells were lysed, and appropriate dilutions of mycobacteria were plated onto MB7H11 agar to determine the CFU.

### Metabolite Extraction

Samples for metabolite analysis were prepared as described previous (45). Briefly, 1 ml of 50% methanol was added to 100 mg of *Mtb*-infected lung tissue and homogenized in a dounce homogenizer to make a uniform suspension. For capillary electrophoresis mass spectrometry (CE-MS), 200 µl of homogenate was mixed with 200 µl of 0.2 M formic acid and vortexed for 2 min. The samples were cleared by centrifugation at 16,000 × *g* for 10 min at 4°C and the supernatant was filter sterilized using 0.22-µm spin X columns (Sigma). For GC-MS, 200 µl of homogenate was mixed with 640 µl of methanol and vortexed for 2 min. 160 µl of MTBE (Methyl-tert-butyl ether); 80:20 (Methanol:MTBE) was added to each sample. Metabolites were then extracted for 1 h with shaking at room temperature, then centrifuged at 4,000 × *g* at 20°C for 20 min. The supernatants were sterile filtered using 0.22-µm spinX columns. All samples were passed through a Millipore filter (30 kDa cutoff) to remove large proteins. Samples were dried and stored at −80°C until analyzed.

### Capillary Electrophoresis Mass Spectrometry (CE-MS)

Dried samples were resuspended in 0.1 mM formic acid containing 0.2 mmol methionine sulfone (as an internal standard) (Sigma-Aldrich, Germany) and vortexed for 1 min followed by centrifugation at 12,600 ×*g* for 15 min. The cleared solution was analyzed by capillary electrophoresis coupled to a mass spectrometer detector (CE-ESI-MS-TOF). A CE System (7100 Agilent) coupled to a time-of-flight mass spectrometer (Agilent 6224) was used. The separation was carried out in a fused-silica capillary (Agilent) (total length, 100 cm; i.d., 50 µm). Separation was under normal polarity with a background electrolyte containing 1.0 M formic acid in 10% (v/v) methanol at 20°C. Sheath liquid (6 µl/min) was methanol/water (1/1, v/v) containing 1.0 mM formic acid with two reference masses to allow correction and higher accurate mass in the MS. Samples were hydrodynamically injected at 50 mbar for 35 s and stacked by injecting background electrolyte at 100 mbar for 10 s. The optimized MS parameters were: fragmentor 150 V, Skimmer 65 V, octopole 750 V, nebulizer pressure 10 psi, drying gas temperature at 200°C and flow rate 10.0 l min. The capillary voltage was 3,500 V.

Data were acquired in positive ESI mode with a full scan from *m/z* 87 to 1,000 at a rate of 1.41 scan/s. The resulting CE-MS data files were cleaned of background noise and unrelated ions by the Targeted Feature Extraction tool with Profinder software (B.06.00, Agilent). Data were extracted using a data mining algorithm based on the software. This software contains a list of standards used with their exact monoisotopic mass, migration time, and molecular formula. Metabolites were previously identified in the samples from wild-type mice by comparison of their migration time and spectra with pure standards. Every comparison between *Fth*^+/+^ and *Fth^−/−^* samples was evaluated by Kruskal–Wallis (KW) analysis of variance (ANOVA) to determine which metabolites discriminated significantly between groups (where Benjamini-Hochberg *p*-values < 0.05 were considered significant). This was performed using in-house routines in the software package Matlab version 9 software (The Mathworks, Inc., Natick, MA, USA).

### Gas Chromatography Mass Spectrometry

Dried samples were resuspended in 450 µl of methanol:water:MTBE 74:10:16, after centrifugation at 12,600 × *g* for 15 min, the supernatants were transferred to vials with inserts and evaporated to dryness in a SpeedVac. The dried extracts were submitted to derivatization by a MPS auto sampler (Gerstel) for GC–MS analysis as described by Denkert et al. (85). Briefly, aldehyde and keto groups were first converted to *O*-methyl oximes by reaction with 10 µl pyridine containing 15 mg/ml *O*-methoxyamine (Sigma-Aldrich, Germany) for 60 min at 70°C. In a second step, hydroxyl and amino groups were trimethylsilylated by reaction with 10 µl of *N, O*-Bis(trimethylsilyl) trifluoroacetamide (Sigma-Aldrich, Germany).

Gas chromatography mass spectrometry analysis was performed on an Agilent Technologies 7890B GC system equipped with a Gerstel MPS auto sampler and an Agilent Technologies 7200 accurate-mass Q-TOF detector equipped with electron ionization. A multimode inlet was used at 230°C with a split ratio set at 1:12 with 9.354 ml/min rate to transfer 1 µl sample vapor onto a 30 m × 0.25 mm × 0.25 µm capillary column (Agilent, Germany). As the carrier gas, helium was used at a flow rate of 0.78 ml/min. Column temperature was 60°C for 1 min and then programmed to increase at a rate of 10°C/min until 325°C which was maintained for 10 min. Total run time was 37.5 min. The MS scan mode was chosen as the acquisition mode, with the mass range of 50–650 amu and an acquisition rate of 10 spectra/s.

The individual analytical fingerprints obtained were deconvoluted using Mass Hunter Unknown Analysis Version B.07.00 (Agilent Technologies). This software also allows the identification by comparing the mass spectrum obtained with the one of a target compound library and, as Fiehn’s library includes retention index, the retention time has also used as an additional criterion. After applying the unknown analysis, a cef file was generated to create a method for the MassHunter Quantitative Analysis Version B.07.00 (Agilent Technologies) exporting a data matrix including compound name, mass, CAS number, formula, RT, and abundances per each compound. Signals derived from column bleed were eliminated, then the abundances were normalized using the mean fold change method of normalization.

Every case group and wild-type comparisons were evaluated by KW ANOVA to determine which metabolites discriminated significantly between groups (where Benjamini–Hochberg *p*-values < 0.05 were considered significant). This was performed using in-house routines in the software package Matlab version 9 software (The Mathworks, Inc., Natick, MA, USA). SIMCA-P + 12.0.1 (Umetrics) were used for PCA plotting.

### Real-Time PCR Analysis

Total RNA was isolated from *Mtb*-infected mouse lungs using an RNeasy Plus mini kit per manufacturers recommendations (Qiagen). Genomic DNA was removed by treatment of RNA samples with RNase-free DNase I (Thermo Scientific) for 15 min at RT followed by 30 min at 37°C. One microgram of total RNA was used to generate cDNA by using the iScript cDNA synthesis Kit (Bio-Rad). Quantitative real-time PCR was performed using iQ SYBR Green Supermix (Bio-Rad). The relative gene expression of CD4 and CD8 was normalized to mouse beta 2 microglobulin as an internal control. Primers used in this study are listed in Table S3 in Supplementary Material.

### Murine Histology and Immunohistochemistry

Mouse lung and liver lobes were fixed in 10% neutral buffered formalin for a minimum of 48 h followed by embedding in paraffin by standard procedures. Paraffin sections were cut at a thickness of 5 µm with a rotary microtome. Sections were stained with H&E to score for pathology. For immunohistochemistry, lung sections were deparaffanized in xylenes, rehydrated in a series of rinses in graded ethanol followed by washing in distilled water. Antigen retrieval was performed at 95°C for 30 min. Sections were allowed to cool slowly, washed in distilled water, and incubated in 3% H_2_O_2_ for 10 min. Sections were blocked in 5% goat serum or 5% donkey serum for FtH or FtL, respectively, at room temperature for 1 h. Primary antibodies were diluted (1:200) in blocking buffer for FtH, #sc-25617 and FtL, #sc-14420 (Santa Cruz Biotechnology) and added to sections overnight at 4°C. Sections were washed three times with PBS-T for 5 min each. Goat anti-rabbit (FtH) and donkey anti-goat (FtL) HRP-conjugated secondary antibodies (Jackson Immuno Research Laboratories; 1:500) were diluted in blocking buffer and added to the sections for 1 h at room temperature. Sections were washed three times with PBS-T for 5 min each. Chromagen substrates (Vector Labs) were mixed per the manufacturer’s instructions and added to sections. Sections were washed in distilled water, dehydrated, and mounted using xylene mounting media.

### Human Lung Tissue Processing

Formalin fixed human diseased and control lung samples were processed routinely in a vacuum filtration processor using a xylene-free method with isopropanol as the main substitute fixative. Prussian blue, visualized as an intense blue, or sea-green color visually identified iron deposition in lung tissue according to the following principal: treatment of iron-loaded lung sections with an acid ferrocyanide solution unmask ferric iron as Fe(OH)_3_. The ferric iron reacts with dilute potassium ferrocyanide to produce an insoluble blue, and/or sea-green compound, ferric ferrocyanide (Prussian blue). Sections were cut at 3 µm, baked at 60°C for 15 min, dewaxed through two changes of xylene and rehydrated through descending grades of alcohol to water. Slides were exposed to filtered (1:1) solutions of 2% aqueous potassium ferrocyanide and 2% HCl for 30 min, rinsed with distilled water, and counter stained using Van Gieson for 5 min. Sections were blotted gently until dry, dehydrated in ascending grades of alcohol, cleared in xylene, and mounted with a mixture of distyrene, plasticizer, and xylene.

### Pathology Analyses

Lung tissue was examined by a registered Anatomical Pathologist with more than 25 years of experience in diagnostic and investigative aspects of anatomical pathology. Tissue specimens are examined using an Olympus BX53 microscope, and assessed using conventional pathology criteria. The Department of Anatomical Pathology laboratories are accredited by the Council for Health Service Accreditation of Southern Africa (COHSASA), an internationally accredited quality improvement and accreditation body for health-care facilities based in Africa.

### Slide Scanning

Murine and human tissue specimens were digitized using a Hamamatsu NDP slide scanner (Hamamatsu NanoZoomer RS2, Model C10730-12) and its viewing platform (NDP.View2). The red, green, and blue color balance was kept at 100% whereas gamma correction was maintained between 0.7 and 2. Brightness (60–110%) and contrast (100–180%) settings vary modestly between slides and depends on staining quality. Resolution is usually ~230 nm/pixel yielding a file size of approximately 2.2 GB. High-resolution scanned images are available upon request, or can be downloaded at: https://www.ahri.org/scientist/adrie-steyn/ (Accessed: June 1, 2018).

### Human Pulmonary TB Specimens and Ethics

The Systematized Nomenclature of Medicine (SNOMED) word and code search engines were employed to access the archive of the Department of Anatomical Pathology, National Health Laboratory Service, Durban, South Africa to identify the first five consecutive resected pneumonectomy specimens in 2017 from patients with active pulmonary tuberculosis. The SNOMED word and code search engines were also employed to access postmortem control lung samples from the same archive. Sections from all lobes of the left and right lungs were obtained from the autopsy archive from five individuals who died soon after traumatic injury without pre-existing lung disease, two that were from adults formed the control cohort. Immediately after surgery, lung tissue was fixed in 10% formalin for 10 days for gross and histopathological appraisal.

Iron quantification (Iron Assay Kit, Abcam) was performed on freshly resected lung tissue from nine TB patients suffering from extensive lung disease (often MDR or XDR *Mtb*). All patients undergoing lung resection for TB had completed a full 6–9 month course of anti-TB treatment, or up to 2 years of treatment for drug-resistant TB. Healthy control tissue was obtained from five lung cancer patients.

### Statistics

All experiments were performed at least in triplicate and the data were expressed as mean ± SEM and analyzed, unless indicated otherwise, using the Student’s *t*-test, in GraphPad Prism 6 (GraphPad Software, Inc.).

## Data Availability

The generated RNAseq datasets for this study can be found in the GEO public functional genomics data repository (GEO accession number GSE112361).

## Ethics Statement

All the mice experimental procedures were approved by the IACUC (Animal Protocol Number APN: 20064) at the University of Alabama at Birmingham. For mice studies we adhered as per national/international regulation of “Public Health Service Policy on Humane Care and Use of Laboratory Animals” (NIH), and “Animal Welfare Act and Animal Welfare Regulations” (USDA). The autopsy pathology arm of the study was approved by the University of KwaZulu-Natal (UKZN) Biomedical Research Ethics Committee (BREC, Class approval study number BCA 535/16). The surgical arm of the study protocol, its associated informed consent documents, and data collection tools were approved by the UKZN BREC (approval number BE 019/13). Written informed consent was obtained for all research subjects. Patients were recruited from King DinuZulu Hospital Complex, a tertiary center for TB patients in Durban, South Africa.

## Author Contributions

Conceptualization: VPR, AA, PKR, and AJCS; investigation: animal studies (VPR, KCC, VS, and JNG), flow cytometry (JFG, TDH, KCC, and VPR), XF assays (VS and VPR), transcriptomic profiling and analysis (AJCS), mass spectrometric and metabolomic analyses (FR-S and CB), murine histopathology and qRT-PCR (VPR), human surgical pathology and specialized staining (PKR, KN), autopsy pathology control lung tissue (PKR, TN), surgery of human lungs (RM), immunohistochemistry (AT), iron quantification in human lungs (MAR) reagents provided (MPS), writing: VPR, JNG, PKR, and AJCS; funding acquisition (AJCS and AA). All authors discussed the results and commented on the manuscript.

## Conflict of Interest Statement

The authors declare that the research was conducted in the absence of any commercial or financial relationships that could be construed as a potential conflict of interest.
