# Supplementary material for: Ferritin H Deficiency in Myeloid Compartments Dysregulates Host Energy Metabolism and Increases Susceptibility to Mycobacterium tuberculosis Infection
Source: Front Immunol. 2018 May 3;9:860. doi: 10.3389/fimmu.2018.00860 (PMC5943674; doi:10.3389/fimmu.2018.00860)
Supplement: Supplementary file 1 [file table_1.docx]

**Table S1.** MetPa analysis showing the list of enriched pathways in lungs of *Fth*^-/-^ mice compared to *Fth^+/+^* at 9 weeks post infection.

| **Enriched Pathways and Metabolites** | **KEGG No** |
| --- | --- |
| **Argnine, Proline Metabolism** |  |
| Citrulline | C00327 |
| Argnine | C00062 |
| S-Adenosylmethionine | C00019 |
| L-Proline | C00148 |
| Creatine | C00300 |
| Putrescine | C00134 |
| 4-Hydroxyproline | C01157 |
| Spermidine | C00315 |
| Spermine | C00750 |
| **Glutathione Metabolism** |  |
| Glycine | C00037 |
| Glutathione | C00051 |
| Oxidized glutathione | C00127 |
| Pyroglutamic acid | C01879 |
| Putrescine | C00134 |
| Spermidine | C00315 |
| Spermine | C00750 |
| **Histidine Metabolism** |  |
| L-Histidine | C00135 |
| Histamine | C00388 |
| 1-Methylhistidine | C01152 |
| 1-Methylhistamine | C05127 |
| **Cysteine, Methionine Metabolism** |  |
| L-Methionine | C00073 |
| S-Adenosylmethionine | C00019 |
| S-Adenosylhomocysteine | C00021 |
| 5'-Methylthioadenosine | C00170 |
| L-Serine | C00065 |
| **Glycine, Serine and Threonine Metabolism** |  |
| Glycine | C00037 |
| Betaine | C00719 |
| Sarcosine | C00213 |
| Creatine | C00300 |
| L-Serine | C00065 |
| **Phenylalanine, Tyrosine and Tryptophan** |  |
| L-Phenylalanine | C00079 |
| L-Tyrosine | C00082 |
