# Supplementary material for: Ferritin H Deficiency in Myeloid Compartments Dysregulates Host Energy Metabolism and Increases Susceptibility to Mycobacterium tuberculosis Infection
Source: Front Immunol. 2018 May 3;9:860. doi: 10.3389/fimmu.2018.00860 (PMC5943674; doi:10.3389/fimmu.2018.00860)
Supplement: Supplementary file 2 [file table_2.docx]

**Table S2.** Tentatively identified significant metabolites measured by GC-MS and CE-MS in lungs of *Fth*^-/-^ mice compared to *Fth^+/+^* at 9 weeks post infection.

| **Compound** | **RT** | **Mass** | **Technique** | **PC(9F-9W)**  **%** | **FC(9K/9W)** | **p** | **RSD** |
| --- | --- | --- | --- | --- | --- | --- | --- |
| Glyceric acid | 10.69 | 189.08 | GC-MS | 52.23 | 1.52 | *** | 8.37 |
| Fructose | 17.17 | 103.05 | GC-MS | -33.5 | 0.66 | *** | 8.19 |
| Melezitose | 29.8 | 361.17 | GC-MS | 109.08 | 2.09 | *** | 10.73 |
| Pentanoic acid | 8.32 | 145.1 | GC-MS | 89.68 | 1.9 | *** | 4.85 |
| 2-amino-2-methyl-1,3-propanediol | 10.68 | 218.07 | GC-MS | 52.93 | 1.53 | *** | 7.69 |
| Adenosine 5-monophosphate | 26.83 | 230.12 | GC-MS | 416.28 | 5.16 | *** | 16 |
| Methyl-beta-galactopyranoside | 16.9 | 217.11 | GC-MS | -38.19 | 0.62 | *** | 3.86 |
| Ribose | 15.1 | 103.06 | GC-MS | -32.61 | 0.67 | *** | 7.84 |
| Proline | 13.14 | 258.1 | GC-MS | -30.78 | 0.69 | *** | 6.42 |
| Furanose | 17.1 | 103.06 | GC-MS | -35.86 | 0.64 | ** | 5.79 |
| Adenosine | 23.86 | 230.12 | GC-MS | 117.39 | 2.17 | ** | 11.64 |
| Glycolic acid | 7 | 205.07 | GC-MS | -14.66 | 0.85 | ** | 8.38 |
| Allo-inositol | 17.2 | 318.14 | GC-MS | -44.09 | 0.56 | ** | 15.1 |
| Acetylspermidine | 8.65 | 187.17 | CE-MS | -45.08 | 0.55 | *** | 3.57 |
| Adenosine monophosphate | 19.95 | 347.06 | CE-MS | 140.3 | 2.40 | * | 11.01 |
| Arginine | 9.59 | 174.11 | CE-MS | -39.26 | 0.61 | *** | 3.61 |
| Asparagine | 12.84 | 132.05 | CE-MS | -26.59 | 0.73 | *** | 2.02 |
| Cytidine | 12.15 | 243.09 | CE-MS | -25.98 | 0.74 | *** | 3.28 |
| Galactosylhydroxylysine | 11.23 | 324.16 | CE-MS | -29.98 | 0.70 | *** | 2.33 |
| Glutamate | 13.26 | 147.05 | CE-MS | -12.22 | 0.88 | *** | 1.23 |
| Glutamine | 13.11 | 146.07 | CE-MS | -52.51 | 0.47 | *** | 4.7 |
| Glycine | 10.78 | 75.033 | CE-MS | 15.35 | 1.15 | * | 2.3 |
| Homospermidine | 6.72 | 159.17 | CE-MS | -51.68 | 0.48 | *** | 4.99 |
| Hydroxy-proline | 14.26 | 131.06 | CE-MS | 56.83 | 1.57 | * | 5.92 |
| Hypusine | 8.58 | 233.17 | CE-MS | -27.93 | 0.72 | *** | 5.9 |
| Inosine | 19.96 | 268.08 | CE-MS | -28.02 | 0.72 | *** | 4.39 |
| Leucine//Isoleucine | 12.65 | 131.09 | CE-MS | -24.87 | 0.75 | *** | 1.37 |
| Lysine | 9.31 | 146.11 | CE-MS | -28.33 | 0.72 | *** | 4.45 |
| Methyl-lysine | 9.54 | 160.12 | CE-MS | -55.26 | 0.45 | *** | 4.17 |
| N,N,N-trimethyllysine | 9.63 | 188.15 | CE-MS | -33.47 | 0.67 | *** | 5.54 |
| Phenylalanine M+H | 13.34 | 165.08 | CE-MS | -26.13 | 0.74 | *** | 1.35 |
| Phosphonooxy-lysine | 14.41 | 242.07 | CE-MS | -53.85 | 0.46 | *** | 5.41 |
| Proline betaine | 13.78 | 143.09 | CE-MS | 45.31 | 1.45 | * | 1.14 |
| Putrescine | 6.72 | 88.101 | CE-MS | -43.58 | 0.56 | *** | 5.04 |
| Pyroglutamic acid | 13.2 | 129.04 | CE-MS | -30.37 | 0.70 | *** | 2.23 |
| Spermidine | 6.51 | 145.16 | CE-MS | -31.57 | 0.68 | *** | 4.77 |
| Spermine | 6.48 | 202.22 | CE-MS | -22.09 | 0.78 | *** | 4.99 |
| Thiamine monophosphate | 13.08 | 344.07 | CE-MS | -35.44 | 0.65 | *** | 8.56 |

Retention Time (RT), mass of the target ion, percentage of change (PC), fold change (FC), p-value indicated by * (p>0.04), ** (p>0.02), *** (p<0.02) and relative standard deviation (RSD).
