# Supplementary material for: Ferritin H Deficiency in Myeloid Compartments Dysregulates Host Energy Metabolism and Increases Susceptibility to Mycobacterium tuberculosis Infection
Source: Front Immunol. 2018 May 3;9:860. doi: 10.3389/fimmu.2018.00860 (PMC5943674; doi:10.3389/fimmu.2018.00860)
Supplement: Supplementary file 3 [file table_3.docx]

**Table S3.** Primers used for qRT-PCR analysis of CD4 mRNA and CD8 mRNA in lungs of *Fth^+/+^* and *Fth*^-/-^ mice at 4 weeks post infection*.*

| **Gene** | **Primer Sequence (5’ to 3’)** |
| --- | --- |
| CD4 For | GAGAGTTCCCAGAAGAAGATCAC |
| CD4 Rev | AGGCGAACCTCCTCTAATTAATAC |
| CD8 For | GCTCAGTCATCAGCAACTCG |
| CD8 Rev | ATCACAGGCGAAGTCCAATC |
| B2M For | GGTCTTTCTGGTGCTTGTCT |
| B2M Rev | TATGTTCGGCTTCCCATTCTC |
